# Protein Misfolding and Aggregation in the Brain: Common Pathogenetic Pathways in Neurodegenerative and Mental Disorders

**DOI:** 10.3390/ijms232214498

**Published:** 2022-11-21

**Authors:** Aleksandra Ochneva, Yana Zorkina, Olga Abramova, Olga Pavlova, Valeriya Ushakova, Anna Morozova, Eugene Zubkov, Konstantin Pavlov, Olga Gurina, Vladimir Chekhonin

**Affiliations:** 1 Department Basic and Applied Neurobiology, V.P. Serbsky Federal Medical Research Centre of Psychiatry and Narcology, 119034 Moscow, Russia; 2 Healthcare Department, Mental-Health Clinic No. 1 Named after N.A. Alexeev of Moscow, 117152 Moscow, Russia; 3 Department of Biology, Lomonosov Moscow State University, 119991 Moscow, Russia; 4 Department of Medical Nanobiotechnology, Pirogov Russian National Research Medical University, 117997 Moscow, Russia; 5 National University of Science and Technology “MISiS”, Leninskiy Avenue 4, 119049 Moscow, Russia

**Keywords:** proteinopathy, protein misfolding, protein aggregation, autophagy, schizophrenia, depression, DISC-1, NPAS3, stress, endoplasmic reticulum

## Abstract

Mental disorders represent common brain diseases characterized by substantial impairments of social and cognitive functions. The neurobiological causes and mechanisms of psychopathologies still have not been definitively determined. Various forms of brain proteinopathies, which include a disruption of protein conformations and the formation of protein aggregates in brain tissues, may be a possible cause behind the development of psychiatric disorders. Proteinopathies are known to be the main cause of neurodegeneration, but much less attention is given to the role of protein impairments in psychiatric disorders’ pathogenesis, such as depression and schizophrenia. For this reason, the aim of this review was to discuss the potential contribution of protein illnesses in the development of psychopathologies. The first part of the review describes the possible mechanisms of disruption to protein folding and aggregation in the cell: endoplasmic reticulum stress, dysfunction of chaperone proteins, altered mitochondrial function, and impaired autophagy processes. The second part of the review addresses the known proteins whose aggregation in brain tissue has been observed in psychiatric disorders (amyloid, tau protein, α-synuclein, DISC-1, disbindin-1, CRMP1, SNAP25, TRIOBP, NPAS3, GluA1, FABP, and ankyrin-G).

## 1. Introduction

Mental disorders represent a group of brain illnesses, which significantly impair the quality of life and induce substantial social and cognitive abnormalities. These pathologies are one of the leading causes of disability, along with cancer and cardiovascular diseases. It is estimated that mental disorders affect about 800 million people worldwide, representing more than 10% of world total population [1]. Approximately one in five adults suffers from a psychiatric disorder, stemming foremost from depression [2]. Given the lifelong morbidity and the lack of effective treatment, understanding the pathophysiology of mental illnesses is critical.

The neurobiological causes and mechanisms behind psychopathologies are currently poorly understood. First of all, mental disorders are clinically and etiologically heterogeneous: for example, the central hypothesis put forth to explain depression pathogenesis is the monoamine theory, which links the development of depression to a deficiency in biogenic amines, namely serotonin, norepinephrine, and dopamine [3]. At the same time, depression may also be associated with circadian rhythms, body clocks, and other mechanisms [4].

The exact pathophysiology of schizophrenia remains poorly understood. The most commonly advanced theories are the dopamine hypothesis and the glutamate hypothesis [5]. Other theories include the specific dysfunction of interneurons, abnormalities in the immune system, abnormalities in myelination, and oxidative stress. Schizophrenia is described as a neurodevelopmental disorder with no precise boundary, or single cause, and is thought to develop from gene–environment interactions with involved vulnerability factors [6]. The leading psychiatric diagnoses are depression and schizophrenia, which account for about 50% of all cases [2]. Nevertheless, our idea of other mental impairments, clinical and neurobiological mechanisms, also remains ambiguous.

Proteinopathy—protein conformational disorder, or protein misfolding disease—refers to a class of diseases in which certain proteins lose their structural integrity and, thereby, disrupt the function of cells, tissues, and organs of the body. Often, these proteins fail to fold into their normal configuration; in this misfolded state, the proteins can become toxic in some aspect (a toxic gain-of-function), or they can lose their normal function. Proteinopathies include such diseases as the Creutzfeldt–Jakob disease and other prion diseases, Alzheimer’s disease (AD), Parkinson’s disease, amyloidosis, multiple system atrophy, and a wide range of other disorders [7]. In most, if not all proteinopathies, a change in the three-dimensional folding conformation increases the likelihood of a specific protein to bind to itself. In this aggregated form, the protein is resistant to clearance and can interfere with the normal capacity of the affected organs. In some cases, misfolding of the protein results in a loss of its usual function. Because proteins share a common structural feature known as the polypeptide backbone, all proteins have the potential to misfold under some circumstances. However, only a relatively small number of proteins are linked to proteopathic disorders, possibly due to the structural idiosyncrasies of the vulnerable proteins. The likelihood that proteinopathy will develop increases with certain risk factors that promote the self-assembly of a protein. These include destabilizing changes in the primary amino acid sequence of the protein, post-translational modifications (such as hyperphosphorylation), changes in temperature or pH, an increase in the production of a protein, or a decrease in its clearance. Advancing age is a strong risk factor, as is a traumatic brain injury. In the aging brain, multiple proteopathies can overlap [8].

A number of studies have demonstrated that there is a link between mental disorders and neurodegenerative diseases. Quite often, depressive disorders occur in both Parkinson’s disease and AD patients. Moreover, sometimes depression precedes Parkinson’s disease [9]. People with schizophrenia are prone to develop Parkinson’s disease, while parkinsonism can lead to psychoses [10]. Some signs of schizophrenia can also be observed in AD, and some researchers point to the same risk factors for AD and schizophrenia [11]. Since proteinopathy is a determining factor in the pathogenesis of neurodegeneration, it is also necessary to consider the role it plays in mental disorders. Impairment of proteostasis initiating the formation of abnormal or aggregated proteins in the brain may be one of the aspects of pathogenesis not only for neurodegeneration but also for mental diseases, such as schizophrenia, bipolar disorder, and depression. Researchers have not paid sufficient attention to this issue when the mechanisms involved in protein misfolding may be novel therapeutic targets and should not be overlooked. A limited amount of data concerning this problem has been accumulated so far, and there is lack of a complete, systematic review of the issue. Therefore, the purpose of this review is to summarize available data on the proteostasis impairments that characterize mental disorders.

In the first part of this review, the possible mechanisms of protein folding and accumulation in the cell are described, and evidence of the presence of these pathological processes in schizophrenia, bipolar disorder, and depression is offered. The accumulation of protein aggregates may not only be due to genetic mutations and the accumulation of abnormally misfolded protein but also to impaired post-translational modification of proteins due to endoplasmic reticulum (ER) stress. The second part of the review will describe the main proteins whose accumulation in brain tissues was uncovered in cases of mental disorders.

## 2. Pathology of Proteins Modification

### 2.1. The Common Mechanisms of Protein Misfolding and Aggregation

Accumulation of protein aggregates in cells occurs due to the disruption of several physiological mechanisms: disruption to the ER under stress, defects in chaperone proteins, impaired mitochondrial function, and disruption of autophagy processes. All these processes contribute to the emergence of protein misfolding and their accumulation in the cell or to the impairment of the normally occurring deletion of protein aggregates. In this review, we will expand upon each of these mechanisms.

#### 2.1.1. Disruption to the ER under Stress

ER is the most important component of the eukaryotic cell, where post-translational modification of the protein occurs. ER regulates protein synthesis, folding, and post-translational processing. Proteostasis corresponds to a dynamic coordination between proper folding of newly synthesized proteins, its quality control, and degradation mechanisms. Chaperone proteins participate in the process of protein folding and recognize non-native conformations of polypeptides. In order to perform its various functions, the ER effects critical interactions with other organelles—in particular with mitochondria—to regulate proteostasis and send malformed proteins into degradation. Mitochondria and ER are closely linked through the mitochondria-associated membrane (MAM), which represents about 20% of the total membrane. MAM mediates the actual, physical transmission of stimuli between two organelles and also facilitates chemical signaling (Ca++, reactive oxygen species (ROS)), which is becoming increasingly important in understanding cell survival. MAMs are involved in various cellular processes, including the homeostasis of phospholipids, calcium and mitochondrial proteins, inflammation, autophagy, division and fusion of mitochondria, cell adaptation to stressful factors, etc. [12]. All of the abovementioned processes closely interact with each other to maintain proteostasis, ensure proper protein folding, and prevent the accumulation of protein aggregates [13].

Alterations to these interrelated processes can occur as a result of stress. The concept of stress is widely used in different fields of science: psychology, physiology, and sociology. Physiological stress is currently defined as a state of disturbed homeostasis, including both systemic stress and local stress. Specific stimuli or stressors—such as oxidative stress, lack of nutrition, exposure to adverse physical factors, such as temperature, radiation, or noise, exposure to harmful chemicals, psychological stress, and others—can cause physiological stress in mammals. The specificity of most local physiological stresses depends on where the stressors are produced; for example, cardiac stress, mitochondrial stress, and ER stress are stresses that occur only at specific cellular sites. States in which ER homeostasis is disrupted are called ‘ER stress’. The cellular response to ER stress involves the activation of adaptive mechanisms to overcome stress and restore ER homeostasis [14].

Unfolded protein response (UPR) is the process by which correct protein folding and ensuing cell survival are ensured. This system functions under physiological conditions, removing misfolded proteins from the cell. When UPR detects mutant proteins, it promotes their degradation through catabolic pathways using the ubiquitin proteasome system and autophagic lysosomes. UPR-activated cascades lead to adaptive responses, such as ER-associated biogenesis and ER-associated degradation. The system’s functioning provides for the restoration of cell homeostasis, or cell death. When UPR regulatory mechanisms become overwhelmed, the stress sensors launch apoptosis.

The pathophysiological process of proteostasis impairment represents a decompensated ER stress leading to the accumulation of protein aggregates. ER stress functions via four mechanisms. The first pathway of ER stress activation is synthesis of the proteins that prevent the subsequent aggregation of unfolded proteins. The second mechanism is the expression of chaperone genes in order to increase folding capacity. Other ways the system functions are transcriptional induction of UPR component genes for amplification of the ER-associated degradation ability and activation of apoptosis [15]. IRE1α splices XBP-1 mRNA and activates transcriptional factor X-box binding protein 1 (XBP1). XBP-1 protein binds to the promoters of several of the genes involved in UPR and ERAD (ER-assisted degradation), protein quality control, and organelle biogenesis [16]. PERK activation attenuates total protein synthesis through phosphorylation of eukaryotic translation initiator factor 2α. Phosphorylation of EIF2α enables selective translation of ATF4 mRNA, which encodes a transcription factor that induces the expression of the genes involved in antioxidant responses and the amino acid metabolism. In ER-stressed cells, ATF6 is transported to the Golgi apparatus, where it is subjected to site 1 protease (S1P) and site 2 protease (S2P). This releases a cytosolic domain (ATF6f), which controls several UPR target genes. ATF4 regulates the expression of apoptotic genes [17].

Under prolonged stress, decompensation of these mechanisms leads to cell death. ER dysfunction promotes neurodegeneration processes. However, it has been demonstrated that ER stress is also one of the factors leading to neuronal dysfunction in mental illnesses such as schizophrenia, depression, and bipolar disorder. Three effector kinases regulate the cascade pathways inducing the ER response to stress (inositol-requiring kinase-1 (IRE1), protein kinase RNA-like endoplasmic reticulum kinase (PERK), and those activating transcription factor-6 (ATF6)) [15]. These kinases are sensitive to the accumulation of misfolded proteins [18]. Their activation induces a cascade of biochemical reactions, promoting proapoptotic processes. PERK plays a key role in the survival of a cell under ER stress, while the IRE1 pathway regulates ER-induced apoptosis [15]. Transmembrane kinase PERK phosphorylates eIF2a to attenuate translation and increase ATF4 expression, resulting in enhanced transcription of target genes. The transmembrane transcription factor ATF6 translocates into the Golgi apparatus, where it is cleaved by the proteases S1P and S2P, which leads to increased transcription of ER chaperone genes. Transmembrane RNase IRE1 activates the transcription of UPR component genes [13].

Oxidative stress upsets the balance between the pro-oxidants and antioxidants inducing ROS formation. Mitochondria, ER, and peroxisomes are the three major vital intracellular organelles that generate ROS. Decreased antioxidant levels and increased production of reactive species (RS) and ROS eventually lead to oxidative damage to DNA, carbohydrates, enzymes, proteins, and cellular lipids. The accumulation of misshaped proteins in the ER and mitochondria arises partly owing to the disruption in the ROS-generating activity [19]. Increased oxidative stress occurring in neurodegeneration induces the formation of ROS by mitochondria. Mitochondria controls protein quality with the help of the UPR (mt) system. Decreased activity of mitochondrial proteases contributes to the development of cellular stress, enhancing the formation of protein aggregates. This process is associated with UPR (mt). MAMs promote protein homeostasis in mitochondria and regulate mitochondrial dynamics, ensuring cell survival under mild cellular stress. Under severe stress, ER MAMs mediate the mitochondrial pathway of apoptosis [12].

#### 2.1.2. Defects in Chaperone Proteins

Chaperone proteins are closely associated with the ER. Chaperones carry out the three-dimensional conformation of polypeptides and control protein misfolding and aggregation. They also facilitate the formation of functional proteins and their release from the ER. This process can be disrupted by various pathological conditions, such as oxidative stress, inflammation, and a change in the ionic calcium level. As a result, the accumulation of incorrectly formed proteins occurs in the lumen of the ER. ER chaperones, such as heat shock protein 5 (HSPA5), also called 78 kDa glucose-regulated protein (GRP78), perform the refolding of unfolded proteins that accumulate in the ER after such stress [20].

Cell response to stress can be illustrated by the interaction of the sigma-1 receptor (Sig-1R) and the ER chaperone-binding immunoglobulin protein (BiP). An interorganelle signaling molecule, Sig-1R, is a chaperone protein, which regulates the outflow of calcium from the endoplasmic reticulum of neurons into mitochondria [21]. In normal condition, Sig-1R is associated with BiP. When Sig-1R agonists bind to Sig-1R on the ER, it induces BiP dissociation, promoting chaperone activity. BiP facilitates the transduction of stress signals from the ER to the nucleus through numerous receptors, ion channels, kinases, and the regulatory proteins present in the ER, MAM, cytosol, and nucleus. Thus, BiP modulates bioenergetics, the redox balance, UPR, and cytokine signaling. In neurons, it is involved in dendritic growth, synaptogenesis, and long-term potentiation [22,23].

#### 2.1.3. Disruption of Autophagy Processes

Autophagy is a cellular process, which restores homeostasis by direct degradation of cell parts. Three major autophagy pathways mediating the lysosomal degradation of intracellular components have been described in mammalian cells: (i) micro-autophagy, (ii) macro-autophagy, and (iii) chaperone-mediated autophagy [24]. During micro-autophagy, small portions of the cytosol and proteins are enveloped by lysosomes. Cytoplasmic components, whole organelles, viruses, and proteins are delivered to the lysosomal compartment for further degradation [25]. During macroautophagy, a portion of the cytoplasm becomes surrounded by the segregative membrane, which turns it into a cupped phagophore, the process eventually ending in a new vacuole known as an autophagosome. After this, the fusion of the autophagosome with the lysosome proceeds. This process may be inhibited by lysosomal pathway dysfunction or oxidative stress [25]. Chaperone-mediated autophagy is a selective process in which proteins bind to cytosolic chaperones (for example, LAMP-2) for further recognition and translocation through lysosomes [26].

Under normal conditions, a slight activation of autophagy is required to eliminate damaged proteins that naturally occur in a living cell. The accumulated malformed and ubiquitinated proteins are transported to the nucleus, where they form aggresomes, which are cleared predominantly by autophagy [25]. The appropriate level of autophagy helps to avoid the accumulation of old structures inside the cell. Along with the degradation of the damaged proteins, the process destroys impaired subcellular organelles (mitochondria, endoplasmic reticulum, ribosomes, and even synaptic vesicles) [24].

Autophagy effects the degradation of incorrect protein aggregates, membrane elements, and subcellular organelles, and the recycling of the resulting molecular compounds. Two major cascades control autophagy-related genes: (1) phosphatidylinositol 3-kinase activation and (2) mTOR inhibition induced by rapamycin. The intracellular and extracellular signals regulating autophagy are mediated by two complexes: (1) the Atg1/unc-51-like kinase (ULK) complex, which acts downstream of the mammalian target of rapamycin (mTOR) complex 1 (mTORC1) associated with cell growth, transcription, and translation, and (2) the Beclin 1/class III phosphatidylinositol 3-kinase (PI3K) complex [25]. Mitochondria may also be involved in the process of autophagy. This was discovered only 10 years ago, when Hailey D.W. demonstrated the translocation of some mitochondrial proteins, such as the mitochondrial outer membrane protein, into autophagosomes [27,28].

Unlike most cell types, neurons are extremely vulnerable to the disruption of autophagy, which can be responsible for the postmitotic nature of adult neuron cells. Therefore, neurons cannot dilute potential toxic waste products in daughter cells by mitosis [29]. The maintenance of homeostasis in the organism is realized, among other things, through cell renewal, their programmed death, and the proliferation of new cells. However, in the nervous system, the death of neuronal cell precursors occurs predominantly at the embryonic stage; so, neuronal apoptosis is a much more difficult process to regulate. During the embryonic period, there is an excessive proliferation of neuronal progenitor cells and then a significant level of death of these cells. Apoptosis during the development of the nervous system is crucial. Mature neurons forcefully limit the pathway of apoptosis after their development to ensure their prolonged survival and maintenance throughout life. These restrictions increase the thresholds of sensitivity to apoptosis throughout neuronal differentiation and maturation [30]. When a slight disruption to the autophagy pathway occurs, a cell’s survival of the accumulation of damaged proteins for a long period of time becomes detrimental. In fact, this can lead to the disruption of various cell activities and even to toxicity. When the production of impaired structures exceeds reduced autophagy activity, abnormal proteins accumulate and become visible over the years as intracellular deposits containing altered protein aggregates. Thus, defect of autophagy, eventually, could lead to slowly developing inclusions in neurons. This is facilitated by the intrinsic nature of specific proteins, such as α-synuclein, which is prone to aggregation, since 30% of its native form undergoes spontaneous oligomerization regardless of metabolic conditions [29].

**Figure 1:**
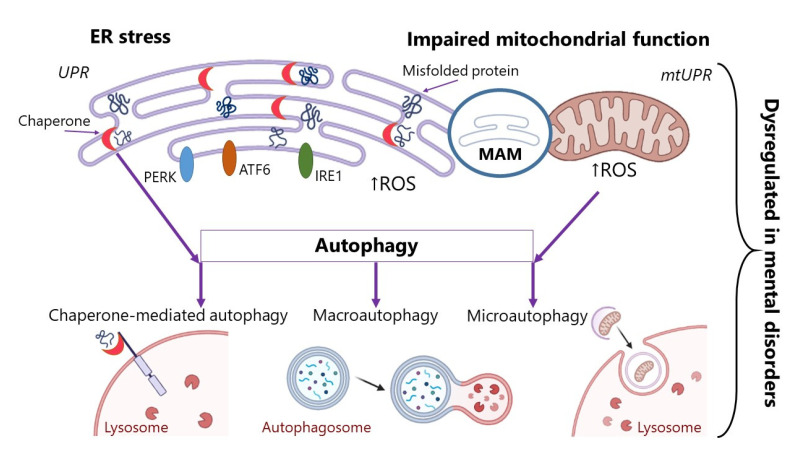
Mechanisms of impaired post-translational modification of proteins: (1) ER stress. The EP response to stress is regulated by three effectors inositol-requiring kinase-1 (IRE1), protein kinase RNA-like endoplasmic reticulum kinase (PERK), and the activating transcription factor-6 (ATF6). ER has its own protein quality control system, unfolded protein response (UPR); (2) disruption in the chaperone proteins involved in folding proteins; (3) impaired mitochondrial function. Mitochondria have their own protein quality control system, unfolded protein response (mtUPR); (4) disruption of autophagy processes. MAM—mitochondria-associated membrane; ROS—reactive oxygen species.

In this section, we have discussed several of the important mechanisms of protein misfolding and aggregation. The summary mechanisms described in this chapter are shown in Figure 1. These alterations can accompany various pathologies [31], but in our review we focus on diseases of the nervous system. We will not dwell on the processes of ER stress in AD, the Parkinson’s and prion diseases, and other neurodegenerations, since many articles and studies have been devoted to these issues [32,33,34]. However, the problem remains very poorly described vis-a-vis mental disorders. There is a significant lack of research on the role of ER stress and UPR activation in mental disorders. In the following section, the physiological mechanisms that may lead to protein aggregation will be discussed in more detail in the context of mental disorders. The features of ER stress, impairment of the chaperone protein’s functioning, and autophagy processes in mental disorders will be described. We believe that this can be one of the aspects of mental disorders’ pathogenesis and that the search for new therapeutic targets could also be informed by UPR systems impairment.

### 2.2. Mechanisms of Protein Misfolding and Aggregation in Mental Disorders

Mental disorders are accompanied by abnormalities in all four physiological mechanisms of protein aggregation: ER stress, a defect in chaperone proteins, impaired mitochondrial function, and impaired autophagy processes. Moreover, many of the available psychotropic drugs target ER and its response to stress [35]. The proposed cascade of events occurring during mental disorders development is the progress of severe or persistent ER stress associated with an increase in ROS and Ca++, which causes upregulation of transcription factors (CHOP) and kinases (JNK) through the activation of the pro-apoptotic branch of UPR. Failure of the UPR leads to the accumulation of protein aggregates. This accumulation causes a stimulation of the inflammasome and provokes increased inflammatory signaling through pro-inflammatory cytokines. After that, the activation of proapoptotic caspases occurs and induces increased apoptosis of neurons [36].

#### 2.2.1. Stress in the Endoplasmic Reticulum

ER stress and oxidative stress are interrelated events that lead to neuronal apoptosis and, ultimately, to cell death in schizophrenic patients. In schizophrenia, the dysregulation of the processing pathways for ER proteins and proteins associated with protein folding, ER quality control (ERQC), and ER-associated degradation (ERAD) occurs. Post-mortem research demonstrated increased expression of proteins associated with the recognition and modification of misfolded proteins. Measurement of protein expression in the dorsolateral prefrontal cortex of patients with schizophrenia revealed an increase in UGGT2, which recognizes the proteins required for further processing and directs them back to the CNX/CRT cycle, and an increase in ER-specific mannosidase EDEM2, which reconstructs terminally malformed glycoproteins and targets them at ERAD. These authors suggest that these changes may reflect potential mechanisms of the abnormal expression of the neurotransmitter-associated proteins previously observed in schizophrenia [37].

Defective ER function is observed in damaged neuronal cells and is associated with the activation of the PERK-phosphorylated ER stress marker. Olanzapine reduces the level of p-PERK in neuronal cells and, thus, attenuates ER stress [38]. The researchers established the existence of some interaction between DISC-1 and ATF4 in the PERK pathway. Trinh et al. discovered that PERK and ATF4 were significantly reduced in the frontal cortex of patients with schizophrenia compared to the control group [39]. The same authors revealed the role of PERK in cognitive dysfunction in mice. In the prefrontal cortex of PERK-deficient mice, eIF2α phosphorylation and ATF4 expression were reduced and associated with increased behavioral perseveration, decreased pre-pulse inhibition, decreased fear extinction, and impaired behavioral flexibility [39]. ER stress inhibitors, such as 4-phenylbutyric acid, salubrinal, taurosodeoxycholic acid, sephin-1, and cordycepin, may be promising as drugs for ER stress alleviation in schizophrenia [36]. Antipsychotic therapy also affects three signaling pathways involved in the ER stress response (PERK, ATF6, and IRE1 pathways).

Studies of bipolar disorder (BD) using patient-derived B-lymphocytes or peripheral blood leukocytes have revealed abnormal ER response to induced stress. So et al. showed that disease progression increases EP’s vulnerability to stress [40]. In BD, a compensatory response of cells to ER stress was impaired, which limited their survival under stressful conditions. The most commonly used mood stabilizers, lithium and valproate, are protective, because they increase the resistance of cells to ER stress [41]. Lithium, widely used in medical treatment, influences the expression of the genes that help to maintain ER function. This bolsters the suggestion that ER stress and UPR processes are important targets in the treatment of mental disorders [42]. Key measures of the ER stress response, such as BiP (chaperone ER), ER-degradation-enhancing alpha-mannosidase-like protein 1 (EDEM1), CHOP C/EBP homologous protein, and X-box binding protein 1 (XBP1), are significantly elevated in patients with a major depression disorder [43]. The XBP1 or CHOP mRNA level, which is raised by ER stress was significantly lower in patients with bipolar disorder [44]. Chronic administration of valproate increases the ER stress protein level in the frontal, parietal cortex, and hippocampus [45].

Some ER stress-related polymorphisms are associated with mental illness. An association of -116C/G polymorphism in the XBP1 gene (X-box binding protein) was observed in patients with schizophrenia [41,46]. In the Polish population, polymorphisms of the HSPA1A, HSPA1B, and HSPA1L genes were found in patients with paranoid schizophrenia [47]. In a study of the Finnish population, physical and social anhedonia was associated with the CRMP1 mediator protein 1 collapsin locus located on the DISC-1 gene. Altered CRMP1 immunoreactivity manifested itself as an increase in CRMP1 in lymphoblastoid cell lines derived from patients with schizophrenia. Thus, this suggests that CRMP1 is a malformed protein that could become a diagnostic marker for schizophrenia [19,48].

#### 2.2.2. Chaperone Proteins

Some studies have shown the involvement of the chaperone protein pathology in psychiatric disorders. Sig-1R is associated with the pathophysiology of many neuronal diseases, such as AD, depression, and schizophrenia [49]. The level of Sig-1Rs is significantly reduced in the brain of patients with schizophrenia [50]. Moreover, Sig-1R represents a target for a range of psychotropic drugs [51]. Receptor stimulation reduces depressive-like behavior in animal models [19]. Deficiency of Sig1R in motor neurons causes ER stress and, therefore, affects mitochondrial dynamics and function [12]. Lithium and valproate may exert their neuro-protective effects by an induction of the brain-derived neurotrophic factor (BDNF) production, as well as heat shock proteins and Bcl-2 [52].

For the diseases associated with impaired protein folding, influencing the activity of chaperone systems may be one of the targets in drug therapy [53], either by altering chaperone gene activity [54] or by acting on chaperones with the molecules that enhance or weaken their function [55]. These therapeutic strategies are being developed for neurodegenerative diseases, among others [54,55,56,57,58].

#### 2.2.3. Autophagy

Lysosomal dysfunction accompanies many neuropsychiatric diseases. Accumulated protein aggregates interpreted as dangerous are associated with molecular patterns and provide for inflammasome activation, leading to inflammatory immune signaling induction and ultimately to increased neuronal apoptosis. Some neuropsychiatric disorders affect autophagic vacuoles (autophagosomes or autophagolysosomes) accumulation, resulting in lysosome dysfunction. Due to disruptions in the autophagic pathway, malformed proteins and aggregates cannot be eliminated from cells, and this leads to cell death. Because of the autophagy system impairments, protein aggregates accumulation eventually overwhelms the cellular degradation and transport systems. These disruptions may also explain the increase in oxidative stress level [12].

Evidence of impaired autophagy in mental illness has been uncovered. The first evidence of system dysregulation in schizophrenia was obtained in 2011 by Horesh’s group, who conducted a gene expression profiling analysis in post-mortem brain samples. The study documented profound differences of expression in Brodmann’s area 22 (BA 22), which is associated with positive schizophrenia symptoms, specifically with auditory verbal hallucinations or “hearing voices”. The vast majority of abnormally expressed genes were related to key autophagy genes (BECN1, ULK2, ATG3), and they were significantly fewer compared to the control group [59].

Progressive synaptic disruption presumably contributes to neurodegeneration in schizophrenia. The mTOR signaling pathway is associated with schizophrenia pathogenesis as well as with extrapyramidal adverse reactions to antipsychotic drugs, which also may be mediated by impaired mTOR-dependent autophagy. Autopsy studies of the brains of schizophrenia patients have revealed the presence of inclusions in neurons that may occur due to the dysfunction in mTOR-related cellular clearance systems [12]. A decrease in the mRNA level of a key protein necessary for autophagy initiation, Beclin 1, has also been documented in the hippocampus of patients with schizophrenia [60]. Suppression of DISC-1 in neurons has been shown to result in excessive activation of Akt signaling. This can be prevented by inhibition of the mTOR target of Akt [61].

Some drugs used to treat schizophrenia also affect autophagy [62]. Olanzapine is considered an mTOR inhibitor and autophagy inducer [63]. Sertindole is a potent inducer of autophagy in neuroblastoma cells [64]. Clozapine activates the autophagy process via the AMPK-ULK1-Beclin1 pathway, as evidenced by increased levels of autophagy markers, raised phosphorylation of AMPK and its subsequent substrates, ULK1 and beclin1, and an increased number of autophagosomes in the frontal cortex. In most studies, the induction of autophagy by antipsychotics was confirmed only when measuring the degradation of autophagy-dependent substrates. Thus, it was shown that chlorpromazine provides for autophagy through the Akt/mTOR pathway inhibition in glioma cells [65]. Lithium indirectly induces autophagy in a mouse model through inositol monophosphatase (IMPA1) and other enzyme suppression in the phosphatidylinositol pathway [66]. Recently uncovered evidence supports mTORC1 disruption in depression [67]. Fast-acting antidepressants affect a variety of receptors, their subunits, and sites, including NMDA, AMPA, m1ACh, mGluR2/3, and GluN2B, to enhance mTOR function, resulting in a rapid antidepressant effect [68]. Existence of variants of the AKT1 gene in patients with schizophrenia supports the idea of the key role played by impaired Akt-mTOR signaling in the pathogenesis of this psychiatric disorder [12]. Ablation of the genes atg5 and atg7 required for autophagy leads to the accumulation of ubiquitin-positive aggregates and a progressive loss of neurons in mice [69].

#### 2.2.4. Therapeutic Approaches

Crucially, determining ways of possible therapeutic intervention in the described mechanisms of dysregulation is necessary. At the moment, there is not much research directed at developing therapeutic strategies that take aim at the protein clotting system, influence ER stress components, the chaperone protein system, and autophagy, specifically for psychiatric, but not neurodegenerative, diseases. These pathways can be affected by various therapeutic interventions, such as antioxidants, sigma-1 receptor agonists, and gene therapy. The use of synthetic and natural ER-stress inhibitors, such as 4-phenyl butyric acid or salubrinal, is also promising [19,49]. It can be hypothesized that a therapeutic intervention of the type could prevent or stop the formation of protein aggregates in brain tissue, which would contribute to the recovery of patients afflicted with psychiatric disorders.

## 3. Aggregating Proteins in Neurodegenerative and Mental Disorders

Impaired proteostasis is well documented in neurodegenerative diseases, and the accumulation of specific proteins, such as amyloid, tau, and α-synuclein, is a hallmark of these pathologies. As relates to mental disorders, no aggregating proteins with such a significant role in the pathogenesis have been identified so far. However, enough studies demonstrate the possibility of a pathological aggregation of certain proteins in brain tissues during mental disorders. Potentially, these proteins may be involved in the development of mental diseases. In this section, we will primarily focus on the role of known aggregating proteins associated with neurodegeneration in psychiatric disorders and then move on to lesser-known pathological protein aggregates that have been identified in psychiatric illnesses.

### 3.1. Amyloid

Beta-amyloid forms from its transmembrane protein precursor by proteolytic cleavage. APP is essential to neuroplasticity, the formation of new synapses, and overall viability of neurons [70]. Beta-amyloid is found in amyloid plaques in AD. Some evidence points to the presence of amyloid in the brains of schizophrenic patients, but the exact role of amyloid in the pathogenesis of schizophrenia is unclear. Amyloid level assessment in the cerebrospinal fluid has demonstrated no association between amyloid and cognitive impairment in patients with schizophrenia. However, no data on any association of cognitive impairment with the level of amyloid in the cortical region of the brain have been obtained [71]. Nevertheless, various animal studies have demonstrated that direct introduction of amyloid into brain structures results in behavioral disruption in animals.

Cognitive impairment and memory deficits are directly related to the progressive accumulation of beta-amyloid plaques. Clinical studies have shown that beta amyloid is associated with anxiety and depressive disorders [72,73,74,75]. Supporting data have also been obtained in preclinical research. EPM (elevated plus maze) is the most widely used test in behavioral analyses of rodents aimed at detecting the anxiolytic and anxiogenic properties of the drugs used. A beta-amyloid injection was found to significantly reduce OAT (time in the open arm/time in Bopen + closed arm) in the EPM, by approximately 71.3% compared to the control group. Depressive responses in rats were assessed using the FST (forced swimming test) in the experiment. The depression index is the time of immobility. Thus, a higher immobility time indicates a deeper depressive state, and a lower immobility time indicates a less depressive state. Injection of beta-amyloid significantly increased the immobility time in the FST, by 5.4 times compared with the control group. Swimming time was significantly less by approximately 71.3% in the Aβ group compared to the controls [76].

The pathogenesis of anxiety and depression is strongly associated with oxidative stress. Oxidative stress leads to molecular damage, inflammation due to the induction of specific cytokines, impaired neurogenesis, and altered neural signaling pathways. Antioxidant and proteolytic systems serve to protect against oxidative stress and against neurons damage, generally. This process is realized through autophagy, whose impairments are associated with neuropsychiatric disorders. In a study by Nayereh Zare, Geldanamycin protected against memory deficits by modulation of Aβ-induced oxidative stress and apoptosis [76]. In addition, Bilici and colleagues suggested that increased levels of MDA, a biomarker of oxidative stress, may be an inducer of depressive behavior in beta-amyloid-treated rats [77].

Soluble Aβ42 aggregates exhibit the highest neurotoxicity among all forms of amyloid, causing oxidative stress and cognitive impairment, and increasing anxiety. This effect is associated with a facilitated expression of connexins, such as Cx43, and a chronic release of glutamate and ATP in the surrounding astrocytes. Both of these factors are connected with raised levels of anxiety and stress. For example, Sheetal Sharma and colleagues induced anxiety behavior and the effects of oxidative stress in rats by single intracerebral ventricular (icv) injection of Aβ42 oligomers. At the same time, carbenoxolone, a gap junction blocker, prevented the oxidative damage and anxiety-like behavior and decreased the expression of Cx43, perhaps by preventing the release of small neurotoxic molecules [78].

The neurotoxicity associated with amyloid and tau aggregation may represent a pathophysiological cascade that, along with vascular disorders, may predispose individuals to apathy and depression in later life. This condition results in poor functioning. It is widespread and difficult to treat with antidepressants. A neuroimaging study by Harris A. Eyre and colleagues showed that apathy in depression in later life is associated with higher levels of amyloid and/or tau in the anterior cingulate cortex [79].

The relationship between the accumulation of beta-amyloids in the brain and the level of depression over time was studied by Nancy J. Donovan and colleagues. The cohort of patients represented a group of elderly people with normal cognitive performance. In older people with higher levels of beta-amyloid, an increase in anxiety-depressive syndromes over time was revealed. A prior diagnosis of depression was associated with more severe, but not deteriorating, symptoms. It can be assumed that the aggravation of anxiety-depressive symptoms may be directly or indirectly associated with an increase in the beta-amyloid level [80].

### 3.2. Tau Protein

Neurofibrillary tangles represent important pathological correlates of clinical symptoms. Neurofibrillary tangles are predominantly composed of the hyperphosphorylated tau protein. Tau belongs to the microtubule-associated proteins (MAPs) family. Its role is very important in the stabilization of microtubules, which are involved in kinesin and dynein-based anterograde and retrograde transport. Disruption in axonal transport leads to mistakes in the work of synapses. Moreover, tau is related to mitochondrial function and deficiencies in oxidative phosphorylation or apoptotic activity [81]. Intracellular involute tau fibers disrupt axonal transport between the cell body and multiple synapses, which is critical to neuronal functioning and survival. In AD, tau protein axonal localization reflects the degree of neurofibrillary pathology and neurodegeneration. Abnormal and phosphorylated tau accumulation is mostly significant in patients with dementia. At the same time, there is a dearth of research involving patients with schizophrenia [82].

Mukaetova-Ladinskaya and colleagues uncovered an increased concentration of the tau protein in extracts of the gray matter of the cerebral cortex. Their data suggest that the amount of soluble β-amyloid (Aβ) and abnormal levels of the tau protein may be enhanced in AD patients who suffer from psychosis. The increased concentration of phosphorylated tau (p-Tau) in the dorsolateral prefrontal cortex (DLPFC) in AD patients with psychosis could be a potential sign of facilitated kinase activity and subsequent tau phosphorylation. This research indicates more pronounced tau pathology in patients with AD and psychosis [83].

Elevated levels of total tau and p-Tau in the cerebrospinal fluid are generally considered to be sensitive markers of neurodegeneration in AD. Schönknecht and colleagues studied taupathy in the cerebrospinal fluid (CSF) of schizophrenic patients and uncovered an absence of raised levels of tau in the CSF [84]. Another recent study by Frisoni and colleagues investigated tau protein levels in the CSF of elderly patients with schizophrenia. It showed elderly patients with normal levels of tau. The authors suggested that in that context, the tau protein concentration did not correspond to neurodegeneration and could have been secondary to the effects of drug treatment [85]. Ömer Faruk Demirel and colleagues measured total tau protein and phosphorylated tau in the serum of patients with schizophrenia. The results of the study showed levels of total tau and p-Tau in the serum of patients that were significantly lower compared to those of healthy people. The research also revealed a positive correlation between total tau levels and the number of previous electroconvulsive therapy (ECT) sessions in the patient group. Nevertheless, no correlation was established between the PANSS scores and tau levels or between smoking and antipsychotic drug use and the tau concentration [82]. If we compare the tau protein content in patients with AD, we can observe a similar pattern for CSF: there is also an increase in the tau protein in CSF in AD [86,87]. Regarding blood, current data suggest a slight increase in plasma tau levels in AD [87], which is not entirely consistent with the blood tau protein in schizophrenia, a phenomenon that requires further study.

The significance of hyperphosphorylated tau in the study of depression pathogenesis remains unclear. Some studies of cerebrospinal fluid and post-mortem specimens of patients with varying degrees of depressive and cognitive symptoms severity have been performed. In particular, Pomara and colleagues uncovered no changes in total or phosphorylated tau in 28 cognitively normal but severely depressed older adults compared to 19 healthy controls [88]. Another research into a post-mortem cohort of 582 older adults revealed a link between depression and the rate of cognitive decline. However, this connection was independent of markers of dementia-related pathology, including Aβ plaques and tau tangle density. Another two, separate post-mortem studies of subjects with AD dementia established a correspondence between comorbid depression and higher levels of tau tangles [89]. The study by Jennifer R. Gatchel and colleagues discovered that more severe depressive symptoms were significantly linked to more pronounced tau in the lower temporal lobe. A similar result was obtained in a model that estimated the relationship between depressive symptoms and tau in the entorhinal cortex. More severe depressive symptoms were moderately associated with higher levels of tau in the lower temporal lobe and, to a lesser degree, in the entorhinal cortex [90]. In 2021, a clinical case that examined a patient that combined multiple extraneural metastases of rectal carcinoma with a suicide attempt, severe depression, and tauopathy was published. The patient exhibited no other mental or neurological disorders. The study revealed marked age-related tau astrogliopathy (ARTAG) of all five types (subpial, subependymal, gray and white matter, and perivascular) affecting the cortical and subcortical regions of the brain. This pathology has been associated with intermediate neuropathological changes in AD, cerebral amyloid angiopathy, α-synuclein proteinopathy with Lewy bodies (Braak stage 4), and proteinopathy associated with the response to multiple systemic transactivation by the 43 kDa DNA-binding protein (TDP-43). It also affected astroglia. Thus, a complex and extensive combination of multiple proteinopathies was identified in the patient. As such a condition was accompanied by severe depressive symptoms, it could potentially be read as a reflection of the association between depression and tauopathy [91]. A better understanding of the association between subclinical depressive symptoms and cerebral tau in vivo in the context of aging is essential for development of more effective preventive services and treatments targeting both depressive symptoms and progressive cognitive decline. This could also help us detect the earliest preclinical changes, which may provide an opportunity for timely intervention.

### 3.3. α-Synuclein

The α-synuclein is a neuronal protein found in presynaptic terminals, predominantly in the neocortex, hippocampus, and substantia nigra. The α-synuclein aggregates accumulate during Parkinson’s disease, but the mechanisms of this neurodegeneration are not fully understood. Parkinson’s disease is often accompanied by depression and anxiety. Depression and anxiety precede the onset of motor symptoms and have a negative impact on the quality of life. Dysfunction of the serotonergic (5-HT) system, which regulates emotions, plays a major role in the development of depressive symptoms. Lluis Miquel-Rio developed a mouse model of α-synucleinopathy in their research. The results of their study showed that α-synucleinopathy in the 5-HT neurons negatively affects the brain circuits that control mood and emotion, resembling the neuropsychiatric symptoms that occur early in Parkinson’s disease. Thus, lowering of α-synuclein levels in the early stages of the disease may help reduce the severity of the depression [92].

The β-synuclein is a presynaptic phosphoprotein that is abundantly expressed in the brain. The role of β-synuclein in physiological processes is not completely clear. It has been suggested that it suppresses the processes triggered by α-synuclein and prevents neurodegeneration [90]. However, Ohtake and colleagues identified two missense mutations of β-synuclein in patients with Lewy body dementia [93]. Later, Masayo Fujita and colleagues developed transgenic mice that expressed P123H β-synuclein and suffered from dementia with Lewy bodies. These mice experienced memory impairment and movement disorders [94].

### 3.4. DISC-1

The DISC-1 gene was first identified in a Scottish family, where t (1; 11) (q42.1; q14.3) translocation mutation carriers were found to have various mental illnesses with approximately 70% penetrance. This mutation led to changes in the DISC-1 and DISC-2 genes, where DISC-2 is a non-coding region with regulatory activity of the DISC-1 protein expression [95]. The DISC-1 gene may be one of the genes responsible for the susceptibility to chronic mental disorders, such as schizophrenia, bipolar disorder, and severe, relapsing depression. The DISC-1 protein is functionally involved in many processes that regulate the development of the nervous system and brain maturation, such as neuronal proliferation, differentiation, migration, cytoskeletal modulation, and post-translational regulation, through various signaling pathways. DISC-1 is a structural protein that localizes in the postsynaptic region of the synapse, as well as in the centrosome, cytoskeleton, mitochondria, and nucleus, and interacts with more than another 200 proteins [95].

The study of the protein’s structure demonstrated the internal irregularity of the N-terminal region of DISC-1 and identified four functional helical and soluble domains in the C-terminal region, named D. I. S. C. The C-terminal region may determine the pathophysiology of mental illness associated with protein assembly and aggregation disorders, since previously discovered mutations for the most part affected this part of the protein [96]. Recently obtained data concerning the characteristics of the C-region of DISC-1 demonstrate that this region is structured and polymorphic, forming oligomers and β-fibrils. As a result of various mutations, truncate variants of the protein appear, which leads to its aggregation and the formation of insoluble forms. Thus, increased expression of DISC-1 can lead to the formation of aggregates [97]. Such DISC-1 aggregates, insoluble even in the presence of detergent sarcosyl, have been found in patients with a chronic mental illness [48].

In vitro studies have revealed that overexpression of the DISC-1 protein results in the formation of large, perinuclear insoluble aggregates that form aggresomes, as evidenced by the colocalization of DISC-1 with aggressive markers, and the dispersion of small and medium-sized aggregates in the cytosol [98]. Aggregation of DISC-1 in vitro can be stimulated by the induced cellular stress caused by hydrogen peroxide treatment [99] and by an increase in dopamine levels [98]. Aggressive aggregates are able to recruit endogenous soluble DISC-1, thereby reducing the functional activity of the protein and can recruit other proteins, such as dysbindin [100]. Aggregation of the transgenic DISC-1 protein also leads to the recruitment of the endogenous protein to the insoluble fraction [101]. One of the consequences of DISC-1 aggregation is the disruption of the mitochondrial transport rate [99]. In addition, it has been shown that aggregates of full-length DISC-1 can be transported to intercellular contacts similar to prion and prion-like proteins using transport through membrane nanotubules (TNTs) [98] and that fragments of the C-terminal region of DISC-1 are directly internalized by other cells [48].

The study of the DISC-1 point mutation in a mouse model of schizophrenia revealed that one of the possible mechanisms of aggregates formation represented an increase in the intermolecular cohesion of the protein. It was demonstrated that the decrease in total or functional DISC-1 led to impaired sensorimotor gating, which often occurs in patients with schizophrenia [102]. Experimental data from a study of transgenic rats confirmed the suggestion that the DISC-1 gene is involved in the processes of postnatal neurogenesis and that, together with environmental factors, it can contribute to the dysregulation of the dopamine system [103]. Overexpression of full-length DISC-1 and the resulting protein aggregation has been found to trigger the development of behavioral and molecular phenotypes that can also be seen in schizophrenia. These phenotypes are associated with disturbances in the dopamine system, such as dramatic increases in high-affinity D2 receptors and increased clearance of extracellular dopamine due to dopamine transporter translocation in the dorsal striatum [101]. In addition, dysfunctional changes in the hippocampal neural networks occur, affecting spatial memory and leading to an impairment of the ability to adapt to new conditions [104].

### 3.5. Disbindin-1

The chronic mental illness risk gene DTNBP1 encodes the dysbindin-1 protein. This protein represents a part of the lysosome-1-associated organelle biogenesis complex (BLOC-1) in the synapse, modulates NMDA receptors and D2 receptors on the membrane surface, and participates in vesicular transport [105]. As a result of alternative splicing, the human DTNBP1 gene produces at least three distinct isoforms of the protein: dysbindin-1A, -1B, and -1C. In vitro studies have demonstrated that only dibindin-1B has the ability to aggregate. These aggregates are invasive (via exosomal transport) and neurotoxic [106]. Studies in transgenic mice revealed that overexpression of dysbindin-1B can lead to its aggregation and also coaggregation with BLOC-1 subunits, exerting a dominant-negative effect on BLOC-1, with subsequent impairment of synaptic vesicle transport [105]. Overexpression of dysbindin-1B can affect the activity of endogenous dysbindin-1A, possibly recruiting it into aggressive formations, which produces neurotoxic effects. Elevated mRNA levels of genes encoding dysbindin-1B have been found in patients with schizophrenia. Moreover, it has been shown that the intron polymorphism identified in the DTNBP1 gene and associated with schizophrenia affects alternative splicing, resulting in increased expression of dysbindin-1B [107].

### 3.6. CRMP1

The collapsin response mediator protein (CRMP) is a family of five homologous cytosolic proteins (CRMP-1-5) that are involved in microtubule regulation. All of them are highly expressed in a growing and adult nervous system, where they play an important role in the development and maturation of neurons [108]. CRMP expression is altered during mental and neurological disorders in humans [109]. In particular, it is known that CRMP1 is a semaphorin 3A signaling molecule, which mediates neuronal signaling in the developing brain and possesses the reelin-dependent regulation of neuronal migration in the cerebral cortex [110,111]. CRMP1 is also involved in Purkinje cell migration [112]. Reelin anomalies have long been associated with schizophrenia or behavioral control abnormalities [113].

There is at the moment a lack of data on the possibility of aggregation of CRMP proteins in brain tissues during mental disorders. The recent research by Bader and colleagues describes an experiment with the construction of antibodies against the pooled insoluble proteome of a post-mortem brain of patients with schizophrenia to identify unique disease-specific epitopes. One of these epitopes has been found on CRMP1 [114]. The presence of CRMP1 in a purified insoluble brain fraction may be associated with co-aggregation with another true aggregating protein, similar to the reconfiguration of soluble dysbindin to insoluble DISC-1 [100]. The short isoform of CRMP1 cannot aggregate by itself; however, co-expression of the short-spliced form of CRMP1 with DISC-1 results in co-aggregation in the insoluble precipitate of DISC-1 aggress. However, the association between aggregated DISC-1 and aggregated CRMP1 was not strong. This suggests that other factors or proteins may also play a role in CRMP1 aggregation. It was also demonstrated that the long isoform of CRMP1 has its own aptitude for aggregation [48]. Thus, CRMP1 could be a novel candidate protein for the investigation of schizophrenia mechanisms, which functionally interact with DISC-1 and intersect with the reelin and DISC-1 pathways [48].

### 3.7. SNAP25 in the SNARE Complex

SNARE is a neuronal protein complex that plays a central role in neuronal signaling. This protein takes part in the process of synaptic vesicles fusion with the presynaptic plasma membrane, which leads to the release of a neurotransmitter. Disruption of this system results in synaptic dysfunction and neurodegeneration [115,116]. In addition, abnormalities in SNARE are associated with a number of psychiatric disorders [117].

The SNARE complex is formed by several major proteins: SNAP25 (synaptosomal-associated protein 25), syntaxin, and synaptobrevin (or VAMP—vesicle-associated membrane proteins) [118]. All SNARE proteins contain alternating hydrophobic and hydrophilic regions, indicating an internal ability for amyloidogenesis [116,119]. The most pronounced aptitude for aggregation was observed for the helical protein SNAP25. SNAP25 interacts with a number of synaptic proteins and forms several complexes that induce conformational changes in the SNARE complex. The three-dimensional model of the SNAP25 protein showed that a large part of the protein is unordered. This feature of SNAP25 is similar to the amyloid-β and α-synuclein protein structures, which are associated with proteopathies, AD, and Parkinson’s disease [116,120]. In addition, it was shown that the changes in the amyloid precursor protein (APP) and amyloid-β contents in a primary cortical cell culture mirror those in the SNAP25 levels [121]. These data suggest that SNAP25 may fold incorrectly and aggregate subsequently. This assumption was confirmed by the work of Ramos-Miguel and colleagues, who showed the presence of homotetrameric SNAP25 aggregates in autopsy samples of the orbitofrontal cortex of patients with schizophrenia and depression. The amount of tetrameric aggregates (~110 kDa) was seven times higher in patients with schizophrenia compared to the controls. The authors hypothesized that presynaptic overstimulation produces biochemical stress in the SNARE complex, leading to misalignment, due to the misfolding of SNAP25 [122]. Another research team demonstrated that SNAP25 can aggregate by itself [123]. Thus, SNAP25 can probably be considered as a link between synaptopathies and proteopathies [117].

Alterations of SNAP25 expression have been observed in several psychiatric conditions, such as schizophrenia and bipolar disorder [124,125]. Moreover, it has been demonstrated that several SNAP25 polymorphisms (rs363050, rs3746544, rs363043) are associated with autism and attention deficit hyperactivity disorder (ADHD) [126,127,128,129]. Possibly, SNAP25 gene polymorphisms are translated into conformational variants that presumably interact with other SNARE proteins and alter the stability of the SNARE complex. The alteration of SNAP25 conformation weakens the interaction of SNARE proteins and subsequently destabilizes the complex, while extremely strong interactions abnormally increase its stability. Both events presumably change the structural organization of the SNARE complex, which impairs its function and ultimately affects neural signaling. It was also demonstrated that post-translational modification disruptions can alter the functional structure of SNAP25 and increase its tendency to aggregate. Thus, abnormal expression of SNAP25 can presumably be a common neuropathological marker of a number of mental and neurodegenerative disorders [116].

Other proteins of the SNARE complex, such as syntaxin and VAMP, are also associated with psychiatric disorders. Similar to SNAP25, these proteins destabilize the SNARE complex and disrupt neuronal signaling [116]. Ramos-Miguel and colleagues observed an increase in protein–protein interactions in the SNARE complex in schizophrenia patients, which induced the manifestation of complex dysfunction due to abnormal interactions of SNARE proteins. It has been shown that schizophrenia is associated with abnormal SNARE complexes formation in the orbitofrontal and anterior cingulate cortices without any altering of protein expression levels. In addition, the main components of the SNARE complex (SNAP25, syntaxin, VAMP) are known to interact abnormally with the ancillary proteins that are involved in the process of presynaptic binding and release in patients with schizophrenia. Immunoprecipitation studies have demonstrated an increased binding affinity of SNAP25 to the Munc18-1 and Cplx1 proteins in the brain of patients with schizophrenia, which may underlie the formation of the aberrant SNARE complex during the disease [122]. Thus, the misfolding of SNARE complexes and alteration of the protein–protein interactions in the synaptic processes scramble the brain physiology stability and induce the development of neuropathology.

### 3.8. TRIOBP

The TRIO and F-actin binding protein (TRIOBP) gene, previously associated with deafness, encodes several different kinds of proteins, each of which plays a role in the modulation of the actin cytoskeleton. The gene undergoes complex alternative splicing and, thus, induces the development of several proteins, including TRIOBP-1, at the 3′ end of the locus, and TRIOBP-4 at the 5‘-end of the locus [130,131]. In addition, the long isoforms, such as TRIOBP-3 and TRIOBP-6, including the TRIOBP-1 and TRIOBP-4 exons, are also formed [132]. TRIOBP-1 (also known as TARA or TAP68) is one of the short isoforms of TRIOBP representing a mostly structured protein. The functions of TRIOBP-1 in the brain are not well understood, but the protein is known to be involved in actin modulation [130,133]. Actin filaments represent one of the key elements of the cytoskeleton vital for various neuronal processes, including cell motility, neuronal differentiation, and intercellular connections [131]. TRIOBP-1 is also required for proper mitosis and cell migration [131]. TRIOBP-4 is another short isoform, completely unordered and expressed primarily in the inner ear. It was shown that this protein has mutant forms that cause deafness [130,131,134]. The long isoforms of TRIOBP combine the functions of the shorter ones and also play an important role in hearing [130,131].

The highest capacity for aggregation has been attributed through observation to TRIOBP-1. TRIOBP-1 has two separate coiled domains: a central and a C-terminal domain. The central domain is responsible for TRIOBP-1 oligomerization and its tendency for aggregation [132]. The exact mechanism powering this process has yet to be determined, but it is likely to be related with the oligomeric condition and/or other components with which it may interact. For example, it has been shown that the E3 ubiquitin ligase HECTD3 can regulate the ubiquitination and degradation of TRIOBP-1 [133]. Presumably, disruption of this process can lead to TRIOBP-1 protein dysregulation and its aggregation [130]. The possibility of TRIOBP-1 aggregation in mental disorders was shown experimentally. The usage of generated monoclonal antibodies to the total insoluble and aggregated protein fraction of a schizophrenic patient’s brain demonstrated that such antibodies recognize TRIOBP-1 but not TRIOBP-4. This allows one to suggest that TRIOBP-1 is present in an aggregated form in the brain of at least some patients [135]. Overexpression of TRIOBP-1 in mammalian cell culture systems or in primary rat cortex neurons was observed to induce the formation of insoluble aggregates of proteins while such a process was not observed for TRIOBP-4. In cultured cells, endogenous TRIOBP-1 aggregation increased during differentiation, which is consistent with the accumulation of aggregated TRIOBP in postmitotic brain neurons. In addition to the full-length TRIOBP-1 with a molecular weight of 70 kDa, aggregation of shorter (45–60 kDa) proteins, representing the helical regions of TRIOBP-1, was also observed [136]. Bradshaw and colleagues demonstrated that aggregated TRIOBP-1 affects cell morphology when expressed in Neuroscreen-1 cells [136]. Therefore, TRIOBP-1 aggregates may directly influence cell development and not only represent a by-product of other processes associated with serious mental illness. Thus, TRIOBP-1 is one of several proteins that appear to form aggregates in cells and insoluble complexes in the brain through specific, though not yet elucidated, cellular processes [130]. Long isoforms TRIOBP, such as TRIOBP-5, can also be expressed in the brain and form aggregates similar to those of TRIOBP-1 in a cell culture. It is therefore possible that aggregation of longer TRIOBP isoforms may also play a role in mental illness pathogenesis. However, this remains to be explored [130,131].

### 3.9. NPAS3

Neuronal PAS domain protein 3 (NPAS3) is a member of the basic helix-loop-helix (bHLH) family of transcription factors. This group of proteins is involved in the regulation of neurogenesis, metabolism, and circadian rhythms [137]. Initially, the association of NPAS3 with schizophrenia was demonstrated in one interesting family case of schizophrenia, which was diagnosed in both mother and daughter. In that case, a chromosomal translocation disrupting the NPAS3 gene was present in both the mother and daughter affected with schizophrenia, which allowed researchers to associate the gene with mental impairment [138,139]. Some genetic studies have also shown a possible association of NPAS3 with severe psychiatric disorders. For example, it was established that specific NPAS3 haplotypes increase the risk of both schizophrenia and bipolar disorder [139]. It is interesting that the NPAS3 gene has been associated with bipolar disorder and that it is also expressed differently in the dorsolateral prefrontal cortex during bipolar disorder [140].

Established mutation in NPAS3 results in the amino acid replacement of valine for isoleucine (V304I). Recently, a small family with this rare V304I mutation was reported to have two members, one suffering from schizophrenia and another from major depressive disorder [141]. NPAS3 itself is prone to aggregation, but the V304I mutation increases this propensity. V304I has been shown to alter transcriptional activity, decrease soluble endogenous NPAS3, and increase insoluble endogenous NPAS3. The amino acid replacement induced by this mutation occurs in a potentially critical region of protein function, which presumably leads to its destabilization [137]. Potentially, protein conformation changes may contribute to the formation of amyloidogenic intermediates [142].

Another recent work has proved the possibility of NPAS3 aggregation in brain tissues. Samardžija and colleagues studied NPAS3 aggregation by investigating a purified, insoluble fraction of human insular cortex homogenates. Full-length NPAS3 was detected in the insoluble fraction of 70% of the samples, indicating that NPAS3 aggregation could be much more common, and that it is not limited to the rare V304I mutation. It has been shown that NPAS3 aggregation can be caused by oxidative stress. Presumably, oxidative stress plays a greater role in this process than the V304I mutation [143]. Oxidative stress is involved in the pathogenesis of many diseases of the central nervous system, including schizophrenia [144]. Moreover, it can also lead to protein misfolding [145]. The authors concluded that a combination of genetic and environmental factors may contribute to NPAS3 aggregation [143].

### 3.10. Aggregates of GluA1 Subunits of AMPA Receptors Caused by the Dysfunction of the CNTNAP2 Protein

CNTNAP2 (Caspr2) is a neuronal transmembrane protein that is a member of the neurexin superfamily. This protein is involved in neuron–glia interactions and the clustering of K+ channels in myelinated axons. CNTNAP2 is present in many neuron compartments, including dendritic spines, axons, and the soma. It participates in the development of spines, synapses, and dendrites and plays a role in the organization of the axolemma, but the exact mechanisms of its contribution in these processes are unknown [146,147,148]. The CNTNAP2 protein is encoded by the CNTNAP2 gene, whose variability has been associated with mental retardation, autism, epilepsy, and schizophrenia [149,150,151,152]. At the same time, post-mortem neuropathological studies have shown an altered density of the dendritic spines on the pyramidal neurons in people with autism and schizophrenia [153,154]. This suggests a possible role for dendritic development impairment in mental disorders with CNTNAP2 pathology.

The presence of autism-associated behavioral phenotypes in Cntnap knockout mice, such as hyperactivity and epileptic seizures, is an indication of the potential role of CNTNAP2 in the pathogenesis of psychiatric disorders [148]. Varea et al. demonstrated that cultured mature neurons from CNTNAP2 gene knockout mice are characterized by a reduced spine density and reduced levels of AMPA receptor GluA1 subunits in the spines. In addition, large cytoplasmic aggregates of GluA1 were found in such knockout neurons, which may have something to do with the CNTNAP2 protein dysfunction. Furthermore, the presence of such aggregates has been shown to be associated with abnormalities in the transport of synaptic proteins [146]. Interestingly, GluA1-containing agresomes have also been found in normally developing rat spinal cord neurons. These agresomes appeared transiently between embryonic day 17 and postnatal day 19 and contained no other AMPA receptor subunits [155]. Until then, the existence of AMPA receptor-containing aggregates had not been reported in humans with psychiatric disorders or in animal models. The possible role of such aggregates in the pathogenesis of mental disorders remains to be determined.

### 3.11. FABP

Lipid metabolism disorders are associated with mental illness. In particular, the peripheral levels of fatty acids, especially the ω3 and ω6 polyunsaturated fatty acids, have been shown to be associated with the pathophysiology of schizophrenia and autism [156,157,158]. Since ω3 and ω6 are extremely lipophilic molecules, chaperone proteins called fatty acid binding proteins (FABP) are required for their intracellular transport. There are three main types of FABP in the brain, namely FABP3, FABP5, and FABP7, which are expressed in specific cell populations [159]. FABP proteins play an important role in the transport of their insoluble ligands to various cell regions, such as the endoplasmic reticulum, mitochondria, and nucleus [160].

Some data indicate that genetic variations in the FABP genes may be involved in the pathogenesis of mental disorders. Shimamoto and colleagues identified several rare polymorphisms of the FABP3, FABP5, and FABP7 genes in schizophrenia and autism. In particular, they showed that two frameshifting variants (FABP3 c.395delA (p.E132fs) and FABP7 .c.239delA (p.N80fs)) are observed exclusively in affected patients. In addition, these two polymorphisms induce the formation of aggregate-like structures in the cytoplasm without penetrating into the nucleus when overexpressed in Neuro2A cells. This fact points to the predisposition of these proteins to aggregation. Certain pathological behavioral changes have also been observed in Fabp3 and Fabp7 knockout mice, which implies a critical role for them in mental illness phenotypes development [160].

The association of FABP3 with α-synuclein aggregates in animal models of Parkinson’s disease reinforces the idea of the involvement of FABP proteins in aggregates’ formation in brain tissues. It was demonstrated that α-synuclein interacts with FABP3 and that this process promotes oligomerization of α-synuclein in model mice [161]. In addition, it was uncovered that α-synuclein aggregation in dopamine neurons is attenuated in FABP3 knockout mice [161]. A histological study of FABP3 expression in tissues obtained from patients with synucleinopathies, patients with AD, and healthy people showed the presence of FABP3 expression in brain tissues and its colocalization with α-synuclein aggregates in the brains of the people with synucleinopathies, but not with amyloid aggregates, β, or p-tau in the brains of those with AD [162]. These data are an indication that FABP3 expression is associated specifically with α-synuclein, which suggests the important role of FABP3 in the formation of pathological protein aggregates in the brain.

### 3.12. Ankyrin-G

Ankyrin-G is a protein that belongs to the ankyrin family. This protein acts as a scaffold, linking plasma membrane proteins to the actin/β-spectrin cytoskeleton, and thus organizing proteins into domains on the plasma membrane [163]. Ankyrin-G plays an important role in many neurobiological processes, including synaptic transmission and synaptic plasticity [164]. Mutations in the ANK3 gene encoding ankyrin-G have also been shown to be associated with neuropsychiatric disorders [165,166]. Ankyrin-G interacts with deubiquitinase Usp9X. Phosphorylation of Usp9X enhances their interaction, reduces ankyrin-G polyubiquitination, and stabilizes ankyrin-G for the maintenance of dendritic spine development [167]. The USP9X gene has also been associated with abnormalities in brain development [168]. A recent study by Yoon and colleagues demonstrated that Usp9X knockout mice are characterized by increased ubiquitination, reduced density of dendritic spines, and ankyrin-G aggregates formation in the mature cerebral cortex of the mice. These biochemical changes were accompanied by hyperactive behavior on the part of the knockout mice. Therefore, this study is demonstration of a possible pathogenetic mechanism of neurodevelopmental disorders entailing possible neuropsychiatric disorders, which includes aggregation of ankyrin-G in brain tissues [167].

Finally, we summarized the data on the proteins that could potentially be involved in the brain aggregation processes in psychiatric disorders in Table 1.

## 4. Conclusions

Many proteinopathies are characterized by cognitive decline, symptoms of anxiety and depression, as well as the positive symptoms, such as visual and auditory hallucinations, associated with dementia and AD. This is an indication of the involvement of protein conformation and aggregation impairments in the pathophysiological mechanisms of the development of not only neurodegenerative diseases but also schizophrenic and anxiety-depressive spectrum disorders. In this review, we have examined the processes leading to the accumulation of the protein aggregates and misfolded proteins found in various mental illnesses.

The accumulation of misfolded and aggregated proteins in the brain of people with mental disorders may be one of the common features in the pathogenesis of schizophrenia, depression, and bipolar disorder, as well as some other pathologies not considered in this review since no information about them is available at the moment. We believe that additional research is needed in the field of pathological protein aggregates studies, which could contribute to a deeper understanding of the pathogenesis of mental diseases and also represent a prospect in the search for new therapeutic targets.

## Figures and Tables

**Table 1:** Summary of data on aggregating proteins in psychiatric disorders.

Protein	Functions	Mental Disorders	Object	Links
Beta-amyloid	Beta-amyloid is formed from its precursor APP (amyloid precursor protein). APP is essential for neuroplasticity, the formation of new synapses, and overall viability of neurons.	Schizophrenia	Human	[71]
		Behavioral disturbances	Animals	[77]
		Cognitive impairment and memory deficits	Human	[72,73,74,75,76,77,79]
		Anxiety and depressive disorders	Human	[80]
		Depression behavior	Rats	[77]
		Anxiety behavior	Rats	[78]
tau protein	Takes part in the stabilization of microtubules and axonal transport, related to mitochondrial function, deficiencies in oxidative phosphorylation or apoptotic activity	Psychosis	Human	[83]
		Suicide attempt, severe depression and	Human	[89]
		Schizophrenia	Human	[82,84,85]
		Depression	Human	[86,87,88]
*α-synuclein*	Regulates synaptic vesicle transport and neurotransmitter release	Depression	Mice	[92]
β-synuclein	Suppresses the processes caused by α-synuclein and prevents neurodegeneration	Dementia with Lewy bodies	Human	[93]
		Dementia with Lewy bodies, memory impairment, and movement disorders	Transgenic mice that expressed P123H β-synuclein	[94]
DISC-1	The DISC-1 protein is functionally involved in many processes that regulate the development of the nervous system and brain maturation, such as neuronal proliferation, differentiation, migration, cytoskeletal modulation, and post-translational regulation through various signaling pathways.	Schizophrenia	Rats	[95,101]
CRMP1	Involved in microtubule regulation, highly expressed in the growing and adult nervous systems, play an important role in the development and maturation of neurons, mediates neuronal signaling in the developing brain, and promotes the reelin-dependent regulation of neuronal migration in the cerebral cortex, involved in Purkinje cell migration	Schizophrenia	Human	[108,109,110,111]
*Disbindin-1*	Represents a part of the lysosome-1-associated organelle biogenesis complex in the synapse, modulates NMDA receptors and D2 receptors on the membrane surface, and participates in vesicular transport	Schizophrenia	Human	[105,107]
*SNAP25 in the SNARE complex*	Plays a central role in neuronal signaling, takes a part in the process of synaptic vesicles fusion with the presynaptic plasma membrane, which leads to the release of a neurotransmitter	schizophrenia and depression	Human	[115,116,122]
*TRIOBP*	The TRIO and F-actin Binding Protein (TRIOBP) plays a role in the modulation of the actin cytoskeleton.	Schizophrenia	Human	[130,131]
*NPAS3*	Is involved in the regulation of neurogenesis, metabolism, and circadian rhythms	Schizophrenia	Human	[137,143,144,145]
*Aggregates of GluA1 subunits of AMPA receptors caused by the dysfunction of the CNTNAP2 protein*	CNTNAP2 (Caspr2) is involved in neuron-glia interactions and clustering of K+ channels in myelinated axons, takes part in the development of spines, synapses, and dendrites and plays a role in the organization of the axolemma	Psychiatric disorders	Human, mice	[146,148]
		Autism	Mice	[155]
*FABP*	Plays an important role in the transport of fatty acids insoluble ligands to various cell regions, such as the endoplasmic reticulum, mitochondria, and nucleus	Two causing aggregation frameshifting variants (FABP3 c.395delA (p.E132fs) and FABP7 .c.239delA (p.N80fs)) were observed exclusively in patients with schizophrenia and autism	Human (aggregation was shown in a cells culture)	[160]
Ankyrin-G	Acts as a scaffold, linking plasma membrane proteins to the actin/β-spectrin cytoskeleton, and thus organizing proteins into domains on the plasma membrane, plays an important role in many neurobiological processes, including synaptic transmission and synaptic plasticity	Hyperactive behavior entailing possible neuropsychiatric disorders	Mice	[163,164]

## References

[1] Triggiani V., Tafaro E., Giagulli V.A., Sabbà C., Resta F., Licchelli B., Guastamacchia E. (2009). Role of iodine, selenium and other micronutrients in thyroid function and disorders. Endocr. Metab. Immune Disord.—Drug Targets.

[2] Institute of Health Metrics and Evaluation Global Health Data Exchange (GHDx). https://vizhub.healthdata.org/gbd-results/.

[3] Heninger G.R., Delgado P.L., Charney D.S. (1996). The Revised Monoamine Theory of Depression: A Modulatory Role for Monoamines, Based on New Findings From Monoamine Depletion Experiments in Humans. Pharmacopsychiatry.

[4] Ménard C., Hodes G., Russo S. (2016). Pathogenesis of depression: Insights from human and rodent studies. Neuroscience.

[5] Howes O., McCutcheon R., Stone J. (2015). Glutamate and dopamine in schizophrenia: An update for the 21^st^ century. J. Psychopharmacol..

[6] Owen M.J., Sawa A., Mortensen P.B. (2016). Schizophrenia. Lancet.

[7] Dugger B.N., Dickson D.W. (2017). Pathology of neurodegenerative diseases. Cold Spring Harb. Perspect. Biol..

[8] Nelson P.T., Alafuzoff I., Bigio E.H., Bouras C., Braak H., Cairns N.J., Mann D.M., Masliah E., McKee A.C., Montine T.J. (2012). Correlation of Alzheimer disease neuropathologic changes with cognitive status: A review of the literature. J. Neuropathol. Exp. Neurol..

[9] Schrag A., Taddei R.N. (2017). Depression and anxiety in Parkinson’s disease. International review of neurobiology. Int. Rev. Neurobiol..

[10] Waddington J.L., Zhen X., O’Tuathaigh C.M. (2020). Developmental genes and regulatory proteins, domains of cognitive impairment in schizophrenia spectrum psychosis and implications for antipsychotic drug discovery: The example of dysbindin-1 isoforms and beyond. Front. Pharmacol..

[11] Winsky-Sommerer R., de Oliveira P., Loomis S., Wafford K., Dijk D.J., Gilmour G. (2019). Disturbances of sleep quality, timing and structure and their relationship with other neuropsychiatric symptoms in Alzheimer’s disease and schizophrenia: Insights from studies in patient populations and animal models. Neurosci. Biobehav. Rev..

[12] Sukhorukov V.S., Voronkova A.S., Baranich T.I., Gofman A.A., Brydun A.V., Knyazeva L.A., Glinkina V.V. (2022). Molecular Mechanisms of Interactions between Mitochondria and the Endoplasmic Reticulum: A New Look at How Important Cell Functions are Supported. Mol. Biol..

[13] Yoshida H. (2007). ER stress and diseases. FEBS J..

[14] Almanza A., Carlesso A., Chintha C., Creedican S., Doultsinos D., Leuzzi B., Samali A. (2019). Endoplasmic reticulum stress signalling–from basic mechanisms to clinical applications. FEBS J..

[15] Xiang C., Wang Y., Zhang H., Han F. (2017). The role of endoplasmic reticulum stress in neurodegenerative disease. Apoptosis.

[16] Sano R., Reed J.C. (2013). ER stress-induced cell death mechanisms. Biochim. Biophys. Acta.

[17] Hetz C., Chevet E., Harding H.P. (2013). Targeting the unfolded protein response in disease. Nat. Rev. Drug Discov..

[18] Gardner B.M., Pincus D., Gotthardt K., Gallagher C.M., Walter P. (2013). Endoplasmic reticulum stress sensing in the unfolded protein response. Cold Spring Harb. Perspect. Biol..

[19] Patel S., Sharma D., Kalia K., Tiwari V. (2017). Crosstalk between endoplasmic reticulum stress and oxidative stress in schizophrenia: The dawn of new therapeutic approaches. Neurosci. Biobehav. Rev..

[20] Sakurai K., Nishiguchi N., Shirakawa O., Nushida H., Ueno Y., Maeda K., Hayashi Y. (2007). Lack of association between endoplasmic reticulum stress response genes and suicidal victims. Kobe J. Med. Sci..

[21] Fukunaga K., Moriguchi S. (2017). Stimulation of the sigma-1 receptor and the effects on neurogenesis and depressive behaviors in mice. Adv. Exp. Med. Biol..

[22] Kudo T., Kanemoto S., Hara H., Morimoto N., Morihara T., Kimura R., Tabira T., Imaizumi K., Takeda M. (2008). A molecular chaperone inducer protects neurons from ER stress. Cell Death Differ..

[23] Smith H.L., Li W., Cheetham M.E. (2015). Molecular chaperones and neuronal proteostasis. Semin. Cell Dev. Biol..

[24] Ryskalin L., Limanaqi F., Frati A., Busceti C.L., Fornai F. (2018). mTOR-related brain dysfunctions in neuropsychiatric disorders. Int. J. Mol. Sci..

[25] Polajnar M., Žerovnik E. (2014). Impaired autophagy: A link between neurodegenerative and neuropsychiatric diseases. J. Cell. Mol. Med..

[26] Klionsky D.J., Emr S.D. (2000). Autophagy as a regulated pathway of cellular degradation. Science.

[27] Hailey D.W., Rambold A.S., Satpute-Krishnan P., Mitra K., Sougrat R., Kim P.K., Lippincott-Schwartz J. (2010). Mitochondria supply membranes for autophagosome biogenesis during starvation. Cell.

[28] Bras M., Queenan B., Susin S.A. (2005). Programmed cell death via mitochondria: Different modes of dying. Biochemistry.

[29] Burré J., Vivona S., Diao J., Sharma M., Brunger A.T., Südhof T.C. (2013). Properties of native brain α-synuclein. Nature.

[30] Hollville E., Romero S.E., Deshmukh M. (2019). Apoptotic cell death regulation in neurons. FEBS J..

[31] Liu C.Y., Schröder M., Kaufman R.J. (2000). Ligand-independent dimerization activates the stress response kinases IRE1 and PERK in the lumen of the endoplasmic reticulum. J. Biol. Chem..

[32] Ghemrawi R., Khair M. (2020). Endoplasmic reticulum stress and unfolded protein response in neurodegenerative diseases. Int. J. Mol. Sci..

[33] Sprenkle N.T., Sims S.G., Sánchez C.L., Meares G.P. (2017). Endoplasmic reticulum stress and inflammation in the central nervous system. Mol. Neurodegener..

[34] Hashimoto S., Saido T.C. (2018). Critical review: Involvement of endoplasmic reticulum stress in the aetiology of Alzheimer’s disease. Open Biol..

[35] Jóźwiak-Bębenista M., Sokołowska P., Siatkowska M., Panek C.A., Komorowski P., Kowalczyk E., Wiktorowska-Owczarek A. (2022). The Importance of Endoplasmic Reticulum Stress as a Novel Antidepressant Drug Target and Its Potential Impact on CNS Disorders. Pharmaceutics.

[36] Muneer A., Khan R.M.S. (2019). Endoplasmic Reticulum Stress: Implications for Neuropsychiatric Disorders. Chonnam Med J..

[37] Kim P., Scott M.R., Meador-Woodruff J.H. (2018). Abnormal expression of ER quality control and ER associated degradation proteins in the dorsolateral prefrontal cortex in schizophrenia. Schizophr. Res..

[38] Kurosawa S., Hashimoto E., Ukai W., Toki S., Saito S., Saito T. (2007). Olanzapine potentiates neuronal survival and neural stem cell differentiation: Regulation of endoplasmic reticulum stress response proteins. J. Neural Transm..

[39] Trinh M.A., Kaphzan H., Wek R.C., Pierre P., Cavener D.R., Klann E. (2012). Brain-Specific Disruption of the eIF2α Kinase PERK Decreases ATF4 Expression and Impairs Behavioral Flexibility. Cell Rep..

[40] So J., Warsh J.J., Li P.P. (2007). Impaired Endoplasmic Reticulum Stress Response in B-Lymphoblasts from Patients with Bipolar-I Disorder. Biol. Psychiatry.

[41] Kakiuchi C., Ishigaki S., Oslowski C.M., Fonseca S.G., Kato T., Urano F. (2009). Valproate, a Mood Stabilizer, Induces WFS1 Expression and Modulates Its Interaction with ER Stress Protein GRP94. PLoS ONE.

[42] ABengesser S., Fuchs R., Lackner N., Birner A., Reininghaus B., Meier-Allard N., Wallner-Liebmann S. (2016). Endoplasmic reticulum stress and bipolar disorder-almost forgotten therapeutic drug targets in the unfolded protein response pathway revisited. CNS Neurol. Disord. Drug Targets.

[43] Nevell L., Zhang K., Aiello A.E., Koenen K., Galea S., Soliven R., Zhang C., Wildman D.E., Uddin M. (2014). Elevated systemic expression of ER stress related genes is associated with stress-related mental disorders in the Detroit Neighborhood Health Study. Psychoneuroendocrinology.

[44] Hayashi A., Le Gal K., Södersten K., Vizlin-Hodzic D., Ågren H., Funa K. (2015). Calcium-dependent intracellular signal pathways in primary cultured adipocytes and ANK3 gene variation in patients with bipolar disorder and healthy controls. Mol. Psychiatry.

[45] Bown C.D., Wang J.F., Chen B., Young L.T. (2002). Regulation of ER stress proteins by valproate: Therapeutic implications. Bipolar Disord..

[46] Chen W., Duan S., Zhou J., Sun Y., Zheng Y., Gu N., Feng G., He L. (2004). A case–control study provides evidence of association for a functional polymorphism −197C/G in XBP1 to schizophrenia and suggests a sex-dependent effect. Biochem. Biophys. Res. Commun..

[47] Kowalczyk M., Owczarek A., Suchanek R., Paul-Samojedny M., Fila-Danilow A., Borkowska P., Kucia K., Kowalski J. (2014). Heat shock protein 70 gene polymorphisms are associated with paranoid schizophrenia in the Polish population. Cell Stress Chaperones.

[48] Bader V., Ottis P., Pum M., Huston J.P., Korth C. (2012). Generation, purification, and characterization of cell-invasive DISC1 protein species. J. Vis. Exp..

[49] Hashimoto K. (2015). Activation of sigma-1 receptor chaperone in the treatment of neuropsychiatric diseases and its clinical implication. J. Pharmacol. Sci..

[50] Mitsuda T., Omi T., Tanimukai H., Sakagami Y., Tagami S., Okochi M., Kudo T., Takeda M. (2011). Sigma-1Rs are upregulated via PERK/eIF2α/ATF4 pathway and execute protective function in ER stress. Biochem. Biophys. Res. Commun..

[51] Tsai S.-Y., Pokrass M.J., Klauer N.R., E De Credico N., Su T.-P. (2014). Sigma-1 receptor chaperones in neurodegenerative and psychiatric disorders. Expert Opin. Ther. Targets.

[52] Chuang D.-M. (2005). The Antiapoptotic Actions of Mood Stabilizers: Molecular Mechanisms and Therapeutic Potentials. Ann. N. Y. Acad. Sci..

[53] Taldone T., Ochiana S.O., Patel P.D., Chiosis G. (2014). Selective targeting of the stress chaperome as a therapeutic strategy. Trends Pharmacol. Sci..

[54] Lee S., Choi B.R., Kim J., LaFerla F.M., Park JH Y., Han J.S., Kim J. (2018). Sulforaphane Upregulates the Heat Shock Protein Co-Chaperone CHIP and Clears Amyloid-β and Tau in a Mouse Model of Alzheimer’s Disease. Mol. Nutr. Food Res..

[55] Liang X., Wu P., Yang Q., Xie Y., He C., Yin L., Yin Z., Yue G., Zou Y., Li L. (2021). An update of new small-molecule anticancer drugs approved from 2015 to 2020. Eur. J. Med. Chem..

[56] Cotrina E.Y., Gimeno A., Llop J., Jiménez-Barbero J., Quintana J., Prohens R., Arsequell G. (2020). An Assay for Screening Potential Drug Candidates for Alzheimer’s Disease That Act as Chaperones of the Transthyretin and Amyloid-β Peptides Interaction. Chemistry.

[57] Wang L., Bergkvist L., Kumar R., Winblad B., Pavlov P.F. (2021). Targeting Chaperone/Co-Chaperone Interactions with Small Molecules: A Novel Approach to Tackle Neurodegenerative Diseases. Cells.

[58] Campanella C., Pace A., Caruso Bavisotto C., Marzullo P., Marino Gammazza A., Buscemi S., Palumbo Piccionello A. (2018). Heat shock proteins in Alzheimer’s disease: Role and targeting. Int. J. Mol. Sci..

[59] Horesh Y., Katsel P., Haroutunian V., Domany E. (2011). Gene expression signature is shared by patients with Alzheimer’s disease and schizophrenia at the superior temporal gyrus. Eur. J. Neurol..

[60] Merenlender-Wagner A., Malishkevich A., Shemer Z., Udawela M., Gibbons A.S., Scarr E., Dean B., Levine J.M., Agam G., Gozes I. (2015). Autophagy has a key role in the pathophysiology of schizophrenia. Mol. Psychiatry.

[61] Kim J.Y., Duan X., Liu C.Y., Jang M.H., Guo J.U., Pow-anpongkul N., Ming G.L. (2009). DISC1 regulates new neuron development in the adult brain via modulation of AKT-mTOR signaling through KIAA1212. Neuron.

[62] Vucicevic L., Misirkic-Marjanovic M., Harhaji-Trajkovic L., Maric N., Trajkovic V. (2018). Mechanisms and therapeutic significance of autophagy modulation by antipsychotic drugs. Cell Stress.

[63] Cui F., Gu S., Gu Y., Yin J., Fang C., Liu L. (2021). Alteration in the mRNA expression profile of the autophagy-related mTOR pathway in schizophrenia patients treated with olanzapine. BMC Psychiatry.

[64] Shin J.H., Park S.J., Kim E.S., Jo Y.K., Hong J., Cho D.H. (2012). Sertindole, a potent antagonist at dopamine D2 receptors, induces autophagy by increasing reactive oxygen species in SH-SY5Y neuroblastoma cells. Biol. Pharm. Bull..

[65] Shin S.Y., Lee K.S., Choi Y.K., Lim H.J., Lee H.G., Lim Y., Lee Y.H. (2013). The antipsychotic agent chlorpromazine induces autophagic cell death by inhibiting the Akt/mTOR pathway in human U-87MG glioma cells. Carcinogenesis.

[66] Gould T.D., O’Donnell K.C., Dow E.R., Du J., Chen G., Manji H.K. (2008). Involvement of AMPA receptors in the antidepressant-like effects of lithium in the mouse tail suspension test and forced swim test. Neuropharmacology.

[67] Cholewinski T., Pereira D., Moerland M., Jacobs G.E. (2021). MTORC1 signaling as a biomarker in major depressive disorder and its pharmacological modulation by novel rapid-acting antidepressants. Ther. Adv. Psychopharmacol..

[68] Athira K.V., Mohan A.S., Chakravarty S. (2020). Rapid acting antidepressants in the mTOR pathway: Current evidence. Brain Res. Bull..

[69] Komatsu M., Waguri S., Chiba T., Murata S., Iwata J.I., Tanida I., Tanaka K. (2006). Loss of autophagy in the central nervous system causes neurodegeneration in mice. Nature.

[70] Tatarnikova O.G., Orlov M.A., Bobkova N.V. (2015). Beta-amyloid and tau-protein: Structure, interaction, and prion-like properties. Biochemistry.

[71] Takamatsu Y., Ho G., Waragai M., Wada R., Sugama S., Takenouchi T., Hashimoto M. (2019). Transgenerational interaction of Alzheimer’s disease with schizophrenia through amyloid evolvability. J. Alzheimer’s Dis..

[72] Harkany T., O’Mahony S., Keijser J., Kelly J.P., Kónya C., Borostyánkői Z.A., Luiten P.G. (2001). β-Amyloid (1-42)-induced cholinergic lesions in rat nucleus basalis bidirectionally modulate serotonergic innervation of the basal forebrain and cerebral cortex. Neurobiol. Dis..

[73] Sun X., Steffens D.C., Au R., Folstein M., Summergrad P., Yee J., Qiu W.Q. (2008). Amyloid-associated depression: A prodromal depression of Alzheimer disease?. Arch. Gen. Psychiatry.

[74] Young J.J., Lavakumar M., Tampi D., Balachandran S., Tampi R.R. (2018). Frontotemporal dementia: Latest evidence and clinical implications. Ther. Adv. Psychopharmacol..

[75] Namekawa Y., Baba H., Maeshima H., Nakano Y., Satomura E., Takebayashi N., Arai H. (2013). Heterogeneity of elderly depression: Increased risk of Alzheimer’s disease and Aβ protein metabolism. Prog. Neuropsychopharmacol. Biol. Psychiatry.

[76] Zare N., Khalifeh S., Khodagholi F., Shahamati S.Z., Motamedi F., Maghsoudi N. (2015). Geldanamycin reduces Aβ-associated anxiety and depression, concurrent with autophagy provocation. J. Mol. Neurosci..

[77] Bilici M., Efe H., Köroğlu M.A., Uydu H.A., Bekaroğlu M., Değer O. (2001). Antioxidative enzyme activities and lipid peroxidation in major depression: Alterations by antidepressant treatments. J. Affect. Disord..

[78] Sharma S., Sharma N., Saini A., Nehru B. (2019). Carbenoxolone reverses the amyloid beta 1–42 oligomer–induced oxidative damage and anxiety-related behavior in rats. Neurotox. Res..

[79] Eyre H.A., Siddarth P., van Dyk K., St Cyr N., Baune B.T., Barrio J.R., Lavretsky H. (2017). Neural correlates of apathy in late-life depression: A pilot [18F] FDDNP positron emission tomography study. Psychogeriatrics.

[80] Donovan N.J., Locascio J.J., Marshall G.A. (2018). Harvard Aging Brain Study. Longitudinal association of amyloid-β and anxious-depressive symptoms in cognitively normal older adults. Am. J. Psychiatry.

[81] Pîrşcoveanu DF V., Pirici I., Tudorică V.A.L.E.R.I.C.A., Bălşeanu T.A., Albu V.C., Bondari S., Pîrşcoveanu M. (2017). Tau protein in neurodegenerative diseases-a review. Rom. J. Morphol. Embryol..

[82] Demirel Ö.F., Cetin I., Turan Ş., Yıldız N., Sağlam T., Duran A. (2017). Total tau and phosphorylated tau protein serum levels in patients with schizophrenia compared with controls. Psychiatr. Q..

[83] Mukaetova-Ladinska E.B., Harrington C.R., Xuereb J., Roth M., Wischik C.M., Bergener M., Finkel S.I. (1995). Biochemical, neuropathological, and clinical correlations of neurofibrillary degeneration in Alzheimer’s disease. Treating Alzheimer’s and Other Dementias: Clinical Application of Recent Research Advances.

[84] Schönknecht P., Hempel A., Hunt A., Seidl U., Volkmann M., Pantel J., Schröder J. (2003). Cerebrospinal fluid tau protein levels in schizophrenia. Eur. Arch. Psychiatry Clin. Neurosci..

[85] Frisoni G.B., Prestia A., Geroldi C., Adorni A., Ghidoni R., Amicucci G., Giannakopoulos P. (2011). Alzheimer’s CSF markers in older schizophrenia patients. Int. J. Geriatr. Psychiatry.

[86] Olsson B., Lautner R., Andreasson U., Öhrfelt A., Portelius E., Bjerke M., Zetterberg H. (2016). CSF and blood biomarkers for the diagnosis of Alzheimer’s disease: A systematic review and meta-analysis. Lancet Neurol..

[87] Blennow K., Zetterberg H. (2018). Biomarkers for Alzheimer’s disease: Current status and prospects for the future. J. Intern. Med..

[88] Pomara N., Bruno D., Sarreal A.S., Hernando R.T., Nierenberg J., Petkova E., Blennow K. (2012). Lower CSF amyloid beta peptides and higher F2-isoprostanes in cognitively intact elderly individuals with major depressive disorder. Am. J. Psychiatry.

[89] Wilson R.S., Capuano A.W., Boyle P.A., Hoganson G.M., Hizel L.P., Shah R.C., Bennett D.A. (2014). Clinical-pathologic study of depressive symptoms and cognitive decline in old age. Neurology.

[90] Gatchel J.R., Donovan N.J., Locascio J.J., Schultz A.P., Becker J.A., Chhatwal J., Marshall G.A. (2017). Depressive symptoms and tau accumulation in the inferior temporal lobe and entorhinal cortex in cognitively normal older adults: A pilot study. J. Alzheimers Dis..

[91] Klotz S., Fischer P., Hinterberger M., Ricken G., Hönigschnabl S., Gelpi E., Kovacs G.G. (2021). Multiple system aging-related tau astrogliopathy with complex proteinopathy in an oligosymptomatic octogenarian. Neuropathology.

[92] Miquel-Rio L., Alarcón-Arís D., Torres-López M., Cóppola-Segovia V., Pavia-Collado R., Paz V., Bortolozzi A. (2022). Human α-synuclein overexpression in mouse serotonin neurons triggers a depressive-like phenotype. Rescue by oligonucleotide therapy. Transl. Psychiatry.

[93] Ohtake H., Limprasert P., Fan Y., Onodera O., Kakita A., Takahashi H., La Spada A.R. (2004). β-synuclein gene alterations in dementia with Lewy bodies. Neurology.

[94] Fujita M., Hagino Y., Takamatsu Y., Shimizu Y., Takamatsu Y., Ikeda K., Hashimoto M. (2018). Early manifestation of depressive-like behavior in transgenic mice that express dementia with Lewy body-linked mutant β-synuclein. Neuropsychopharmacol. Rep..

[95] Korth C. (2012). Aggregated proteins in schizophrenia and other chronic mental diseases: DISC1opathies. Prion.

[96] Yerabham A.S., Mas P.J., Decker C., Soares D.C., Weiergräber O.H., Nagel-Steger L., Korth C. (2017). A structural organization for the Disrupted in Schizophrenia 1 protein, identified by high-throughput screening, reveals distinctly folded regions, which are bisected by mental illness-related mutations. J. Biol. Chem..

[97] Cukkemane A., Becker N., Zielinski M., Frieg B., Lakomek N.A., Heise H., Weiergräber O.H. (2021). Conformational heterogeneity coupled with β-fibril formation of a scaffold protein involved in chronic mental illnesses. Transl. Psychiatry.

[98] Zhu S., Abounit S., Korth C., Zurzolo C. (2017). Transfer of disrupted-in-schizophrenia 1 aggregates between neuronal-like cells occurs in tunnelling nanotubes and is promoted by dopamine. Open Biol..

[99] Atkin T., Kittler J. (2012). DISC1 and the aggresome: A disruption to cellular function?. Autophagy.

[100] Ottis P., Bader V., Trossbach S.V., Kretzschmar H., Michel M., Leliveld S.R., Korth C. (2011). Convergence of two independent mental disease genes on the protein level: Recruitment of dysbindin to cell-invasive disrupted-in-schizophrenia 1 aggresomes. Biol. Psychiatry.

[101] Trossbach S.V., Bader V., Hecher L., Pum M.E., Masoud S.T., Prikulis I., Korth C. (2016). Misassembly of full-length Disrupted-in-Schizophrenia 1 protein is linked to altered dopamine homeostasis and behavioral deficits. Mol. Psychiatry.

[102] Kakuda K., Niwa A., Honda R., Yamaguchi K.I., Tomita H., Nojebuzzaman M., Kuwata K. (2019). A DISC1 point mutation promotes oligomerization and impairs information processing in a mouse model of schizophrenia. J. Biochem..

[103] Inta D., Meyer-Lindenberg A., Gass P. (2011). Alterations in postnatal neurogenesis and dopamine dysregulation in schizophrenia: A hypothesis. Schizophr. Bull..

[104] Kaefer K., Malagon-Vina H., Dickerson D.D., O’Neill J., Trossbach S.V., Korth C., Csicsvari J. (2019). Disrupted-in-schizophrenia 1 overexpression disrupts hippocampal coding and oscillatory synchronization. Hippocampus.

[105] Yang W., Zhu C., Shen Y., Xu Q. (2016). The pathogenic mechanism of dysbindin-1B toxic aggregation: BLOC-1 and intercellular vesicle trafficking. Neuroscience.

[106] Zhu C.Y., Shen Y., Xu Q. (2015). Propagation of dysbindin-1B aggregates: Exosome-mediated transmission of neurotoxic deposits. Neuroscience.

[107] Xu Y., Sun Y., Ye H., Zhu L., Liu J., Wu X., Xu Q. (2015). Increased dysbindin-1B isoform expression in schizophrenia and its propensity in aggresome formation. Cell Discov..

[108] Yamashita N., Goshima Y. (2012). Collapsin response mediator proteins regulate neuronal development and plasticity by switching their phosphorylation status. Mol. Neurobiol..

[109] Quach T.T., Honnorat J., Kolattukudy P.E., Khanna R., Duchemin A.M. (2015). CRMPs: Critical molecules for neurite morphogenesis and neuropsychiatric diseases. Mol. Psychiatry.

[110] Makihara H., Nakai S., Ohkubo W., Yamashita N., Nakamura F., Kiyonari H., Goshima Y. (2016). CRMP 1 and CRMP 2 have synergistic but distinct roles in dendritic development. Genes Cells.

[111] Yamashita N., Uchida Y., Ohshima T., Hirai S.I., Nakamura F., Taniguchi M., Goshima Y. (2006). Collapsin response mediator protein 1 mediates reelin signaling in cortical neuronal migration. J. Neurosci..

[112] Akinaga S., Harada S., Takahashi M., Kaneko A., Kolattukudy P., Goshima Y. (2022). Loss of CRMP1 and CRMP2 results in migration defects of Purkinje cells in the X lobule of the mouse cerebellum. Brain Res..

[113] Knuesel I. (2010). Reelin-mediated signaling in neuropsychiatric and neurodegenerative diseases. Prog. Neurobiol..

[114] Bader V., Tomppo L., Trossbach S.V., Bradshaw N.J., Prikulis I., Leliveld S.R., Lin C.Y., Ishizuka K., Sawa A., Ramos A. (2012). Proteomic, genomic and translational approaches identify CRMP1 for a role in schizophrenia and its underlying traits. Hum. Mol. Genet..

[115] Alten B., Zhou Q., Shin O.H., Esquivies L., Lin P.Y., White K.I., Kavalali E.T. (2021). Role of aberrant spontaneous neurotransmission in SNAP25-associated encephalopathies. Neuron.

[116] Karmakar S., Sharma L.G., Roy A., Patel A., Pandey L.M. (2019). Neuronal SNARE complex: A protein folding system with intricate protein-protein interactions, and its common neuropathological hallmark, SNAP25. Neurochem. Int..

[117] Cupertino R.B., Kappel D.B., Bandeira C.E., Schuch J.B., da Silva B.S., Müller D., Mota N.R. (2016). SNARE complex in developmental psychiatry: Neurotransmitter exocytosis and beyond. J. Neural. Transm..

[118] McNew J.A., Parlati F., Fukuda R., Johnston R.J., Paz K., Paumet F., Rothman J.E. (2000). Compartmental specificity of cellular membrane fusion encoded in SNARE proteins. Nature.

[119] Broome B.M., Hecht M.H. (2000). Nature disfavors sequences of alternating polar and non-polar amino acids: Implications for amyloidogenesis. J. Mol. Biol..

[120] Choi U.B., McCann J.J., Weninger K.R., Bowen M.E. (2011). Beyond the random coil: Stochastic conformational switching in intrinsically disordered proteins. Structure.

[121] Bailey J.A., Lahiri D.K. (2006). Neuronal Differentiation Is Accompanied by Increased Levels of SNAP-25 Protein in Fetal Rat Primary Cortical Neurons: Implications in Neuronal Plasticity and Alzheimer’s Disease. Ann. N. Y. Acad. Sci..

[122] Ramos-Miguel A., Barr A., Dwork A., Rosoklija G., Mann J., Honer W. (2017). SA101. Characterization of Presynaptic SNAP-25 Aggregates in Human Postmortem Brain: A Novel Pathologic Index in Schizophrenia?. Schizophr. Bull..

[123] Ramos-Miguel A., Jones A.A., Sawada K., Barr A.M., Bayer T.A., Falkai P., Honer W.G. (2018). Frontotemporal dysregulation of the SNARE protein interactome is associated with faster cognitive decline in old age. Neurobiol. Dis..

[124] Thompson P.M., Sower A.C., Perrone-Bizzozero N.I. (1998). Altered levels of the synaptosomal associated protein SNAP-25 in schizophrenia. Biol. Psychiatry.

[125] Etain B., Dumaine A., Mathieu F., Chevalier F., Henry C., Kahn J.P., Jamain S. (2010). A SNAP25 promoter variant is associated with early-onset bipolar disorder and a high expression level in brain. Mol. Psychiatry.

[126] Braida D., Guerini F.R., Ponzoni L., Corradini I., De Astis S., Pattini L., Sala M. (2015). Association between SNAP-25 gene polymorphisms and cognition in autism: Functional consequences and potential therapeutic strategies. Transl. Psychiatry.

[127] Guerini F.R., Bolognesi E., Chiappedi M., Manca S., Ghezzo A., Agliardi C., Clerici M. (2011). SNAP-25 single nucleotide polymorphisms are associated with hyperactivity in autism spectrum disorders. Pharmacol. Res..

[128] Kim E., Song D.H., Kim N.W., Sohn I.J., Cheon K.A. (2017). The relationship between the SNAP-25 polymorphism and omission errors in Korean children with attention deficit hyperactivity disorder. Clin. Psychopharmacol. Neurosci..

[129] Safari M.R., Omrani M.D., Noroozi R., Sayad A., Sarrafzadeh S., Komaki A., Taheri M. (2017). Synaptosome-associated protein 25 (SNAP25) gene association analysis revealed risk variants for ASD, in Iranian population. J. Mol. Neurosci..

[130] Bradshaw N.J., Yerabham A.S., Marreiros R., Zhang T., Nagel-Steger L., Korth C. (2017). An unpredicted aggregation-critical region of the actin-polymerizing protein TRIOBP-1/Tara, determined by elucidation of its domain structure. J. Biol. Chem..

[131] Zaharija B., Samardžija B., Bradshaw N.J. (2020). The TRIOBP isoforms and their distinct roles in actin stabilization, deafness, mental illness, and cancer. Molecules.

[132] Riazuddin S., Khan S.N., Ahmed Z.M., Ghosh M., Caution K., Nazli S., Friedman T.B. (2006). Mutations in TRIOBP, which encodes a putative cytoskeletal-organizing protein, are associated with nonsyndromic recessive deafness. Am. J. Hum. Genet..

[133] Seipel K., O’Brien S.P., Iannotti E., Medley Q.G., Streuli M. (2001). Tara, a novel F-actin binding protein, associates with the Trio guanine nucleotide exchange factor and regulates actin cytoskeletal organization. J. Cell Sci..

[134] Shahin H., Walsh T., Sobe T., Rayan A.A., Lynch E.D., Lee M.K., Kanaan M. (2006). Mutations in a novel isoform of TRIOBP that encodes a filamentous-actin binding protein are responsible for DFNB28 recessive nonsyndromic hearing loss. Am. J. Hum. Genet..

[135] Yu J., Lan J., Zhu Y., Li X., Lai X., Xue Y., Huang H. (2008). The E3 ubiquitin ligase HECTD3 regulates ubiquitination and degradation of Tara. Biochem. Biophys. Res. Commun..

[136] Bradshaw N.J., Bader V., Prikulis I., Lueking A., Müllner S., Korth C. (2014). Aggregation of the protein TRIOBP-1 and its potential relevance to schizophrenia. PLoS ONE.

[137] Nucifora L.G., Wu Y.C., Lee B.J., Sha L., Margolis R.L., Ross C.A., Nucifora F.C. (2016). A mutation in NPAS3 that segregates with schizophrenia in a small family leads to protein aggregation. Mol. Neuropsychiatry.

[138] Kamnasaran D., Muir W.J., Ferguson-Smith M.A., Cox D.W. (2003). Disruption of the neuronal PAS3 gene in a family affected with schizophrenia. J. Med. Genet..

[139] Pickard B.S., Malloy M.P., Porteous D.J., Blackwood DH R., Muir W.J. (2005). Disruption of a brain transcription factor, NPAS3, is associated with schizophrenia and learning disability. Am. J. Med. Genet. B Neuropsychiatr. Genet..

[140] Nurnberger J.I., Koller D.L., Jung J., Edenberg H.J., Foroud T., Guella I. (2014). Psychiatric Genomics Consortium Bipolar Group. Identification of pathways for bipolar disorder: A meta-analysis. JAMA Psychiatry.

[141] Yu L., Arbez N., Nucifora L.G., Sell G.L., Delisi L.E., Ross C.A., Nucifora F.C. (2014). A mutation in NPAS3 segregates with mental illness in a small family. Mol. Psychiatry.

[142] Kelly J.W. (1996). Alternative conformations of amyloidogenic proteins govern their behavior. Curr. Opin. Struct Biol..

[143] Samardžija B., Pavešić Radonja A., Zaharija B., Bergman M., Renner É., Palkovits M., Bradshaw N.J. (2021). Protein aggregation of NPAS3, implicated in mental illness, is not limited to the V304I mutation. J. Pers Med..

[144] Emiliani F.E., Sedlak T.W., Sawa A. (2014). Oxidative stress and schizophrenia: Recent breakthroughs from an old story. Curr. Opin. Psychiatry.

[145] Grune T., Jung T., Merker K., Davies K.J. (2004). Decreased proteolysis caused by protein aggregates, inclusion bodies, plaques, lipofuscin, ceroid, and ‘aggresomes’ during oxidative stress, aging, and disease. Int. J. Biochem. Cell Biol..

[146] Varea O., Martin-de-Saavedra M.D., Kopeikina K.J., Schürmann B., Fleming H.J., Fawcett-Patel J.M., Penzes P. (2015). Synaptic abnormalities and cytoplasmic glutamate receptor aggregates in contactin associated protein-like 2/Caspr2 knockout neurons. Proc. Natl. Acad. Sci. USA.

[147] Gordon A., Adamsky K., Vainshtein A., Frechter S., Dupree J.L., Rosenbluth J., Peles E. (2014). Caspr and caspr2 are required for both radial and longitudinal organization of myelinated axons. J. Neurosci..

[148] Peñagarikano O., Abrahams B.S., Herman E.I., Winden K.D., Gdalyahu A., Dong H., Geschwind D.H. (2011). Absence of CNTNAP2 leads to epilepsy, neuronal migration abnormalities, and core autism-related deficits. Cell.

[149] Alarcón M., Abrahams B.S., Stone J.L., Duvall J.A., Perederiy J.V., Bomar J.M., Sebat J., Wigler M., Martin C.L., Ledbetter D.H. (2008). Linkage, association, and gene-expression analyses identify CNTNAP2 as an autism-susceptibility gene. Am. J. Hum. Genet..

[150] Arking D.E., Cutler D.J., Brune C.W., Teslovich T.M., West K., Ikeda M., Chakravarti A. (2008). A common genetic variant in the neurexin superfamily member CNTNAP2 increases familial risk of autism. Am. J. Hum. Genet..

[151] Gregor A., Albrecht B., Bader I., Bijlsma E.K., Ekici A.B., Engels H., Zweier C. (2011). Expanding the clinical spectrum associated with defects in CNTNAP2 and NRXN1. BMC Med. Genet..

[152] Friedman J.I., Vrijenhoek T., Markx S., Janssen I.M., Van Der Vliet W.A., Faas BH W., Veltman J.A. (2008). CNTNAP2 gene dosage variation is associated with schizophrenia and epilepsy. Mol. Psychiatry.

[153] Glantz L.A., Lewis D.A. (2000). Decreased dendritic spine density on prefrontal cortical pyramidal neurons in schizophrenia. Arch. Gen. Psychiatry.

[154] Hutsler J.J., Zhang H. (2010). Increased dendritic spine densities on cortical projection neurons in autism spectrum disorders. Brain Res..

[155] Serrando M., Casanovas A., Esquerda J.E. (2002). Occurrence of glutamate receptor subunit 1–containing aggresome-like structures during normal development of rat spinal cord interneurons. J. Comp. Neurol..

[156] Arvindakshan M., Ghate M., Ranjekar P.K., Evans D.R., Mahadik S.P. (2003). Supplementation with a combination of ω-3 fatty acids and antioxidants (vitamins E and C) improves the outcome of schizophrenia. Schizophr. Res..

[157] Vancassel S., Durand G., Barthelemy C., Lejeune B., Martineau J., Guilloteau D., Chalon S. (2001). Plasma fatty acid levels in autistic children. Prostaglandins Leukot. Essent. Fat. Acids.

[158] Vaneev A.N., Gorelkin P.V., Garanina A.S., Lopatukhina H.V., Vodopyanov S.S., Alova A.V., Erofeev A.S. (2020). In vitro and in vivo electrochemical measurement of reactive oxygen species after treatment with anticancer drugs. Anal. Chem..

[159] Veerkamp J.H., Zimmerman A.W. (2001). Fatty acid-binding proteins of nervous tissue. J. Mol. Neurosci..

[160] Shimamoto C., Ohnishi T., Maekawa M., Watanabe A., Ohba H., Arai R., Yoshikawa T. (2014). Functional characterization of FABP3, 5 and 7 gene variants identified in schizophrenia and autism spectrum disorder and mouse behavioral studies. Hum. Mol. Genet..

[161] Shioda N., Yabuki Y., Kobayashi Y., Onozato M., Owada Y., Fukunaga K. (2014). FABP3 protein promotes α-synuclein oligomerization associated with 1-methyl-1, 2, 3, 6-tetrahydropiridine-induced neurotoxicity. J. Biol. Chem..

[162] Oizumi H., Yamasaki K., Suzuki H., Hasegawa T., Sugimura Y., Baba T., Takeda A. (2021). Fatty Acid-Binding Protein 3 Expression in the Brain and Skin in Human Synucleinopathies. Front. Aging Neurosci..

[163] Bennett V., Healy J. (2008). Organizing the fluid membrane bilayer: Diseases linked to spectrin and ankyrin. Trends Mol. Med..

[164] Smith K.R., Kopeikina K.J., Fawcett-Patel J.M., Leaderbrand K., Gao R., Schürmann B., Penzes P. (2014). Psychiatric risk factor ANK3/ankyrin-G nanodomains regulate the structure and function of glutamatergic synapses. Neuron.

[165] Iqbal Z., Vandeweyer G., van der Voet M., Waryah A.M., Zahoor M.Y., Besseling J.A., Rooms L. (2013). Homozygous and heterozygous disruptions of ANK3: At the crossroads of neurodevelopmental and psychiatric disorders. Hum. Mol. Genet..

[166] Ferreira M.A., O’Donovan M.C., Meng Y.A., Jones I.R., Ruderfer D.M., Jones L., Craddock N. (2008). Collaborative genome-wide association analysis supports a role for ANK3 and CACNA1C in bipolar disorder. Nat. Genet..

[167] Yoon S., Parnell E., Kasherman M., Forrest M.P., Myczek K., Premarathne S., Penzes P. (2020). Usp9X controls ankyrin-repeat domain protein homeostasis during dendritic spine development. Neuron.

[168] Johnson B.V., Kumar R., Oishi S., Alexander S., Kasherman M., Vega M.S., Kohane I.S. (2020). Partial loss of USP9X function leads to a male neurodevelopmental and behavioral disorder converging on transforming growth factor β signaling. Biol. Psychiatry.

